# Interface Engineering of Water-Dispersible Near-Infrared-Emitting CuInZnS/ZnSe/ZnS Quantum Dots

**DOI:** 10.1021/acs.cgd.4c00528

**Published:** 2024-07-18

**Authors:** Patrick Mann, Simon M. Fairclough, Struan Bourke, Mary Burkitt Gray, Laura Urbano, David J. Morgan, Lea Ann Dailey, Maya Thanou, Nicholas J. Long, Mark A. Green

**Affiliations:** † Department of Physics, King’s College London, The Strand, London WC2R 2LS, U.K.; ‡ Centre for Topical Drug Delivery and Toxicology, School of Life and Medical Sciences, University of Hertfordshire, Hatfield AL10 9AB, U.K.; § School of Chemistry, Cardiff University, Main Building, Park Place, Cardiff CF10 3AT, U.K.; ∥ Department of Pharmaceutical Sciences, University of Vienna, Josef-Holaubek-Platz 2, 1090 Vienna, Austria; ⊥ Institute of Pharmaceutical Science, King’s College London, 150 Stamford Street, London SE1 9NH, U.K.; # Department of Chemistry, Imperial College London, Molecular Sciences Research Hub, White City Campus, London W12 0BZ, U.K.

## Abstract

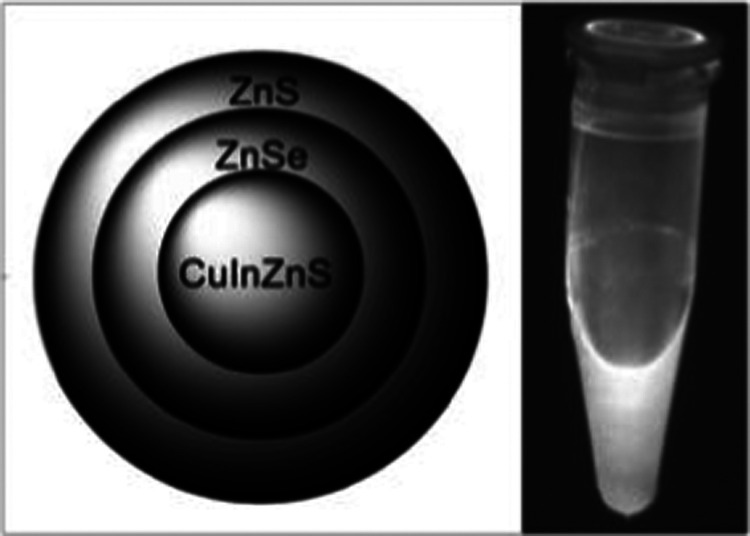

We report the synthesis of near-infrared (IR)-emitting core/shell/shell quantum dots of CuInZnS/ZnSe/ZnS and their phase transfer to water. The intermediate ZnSe shell was added to inhibit the migration of ions from the standard ZnS shell into the emitting core, which often leads to a blue shift in the emission profile. By engineering the interface between the core and terminal shell layer, the optical properties can be controlled, and emission was maintained in the near-IR region, making the materials attractive for biological applications. In addition, the hydrodynamic diameter of the particle was controlled using amphiphilic polymers.

## Abstract 2

In this report, we describe the use of an intermediate ZnSe layer in the synthesis of CuInZnS/ZnS quantum dots to inhibit the migration of shell ions into the core, preserving the core optical properties and impeding the resulting blue shift in optical properties which occurs in samples without this barrier.

## Introduction

Quantum dots (QDs) have emerged over the last 3 decades as superior light emitting materials, notable for covering wavelengths from near-ultraviolet (UV) into the infrared with narrow, bright emission profiles. These materials have found applications in diverse markets, from biological labeling to solar energy harvesting. High-quality crystalline II–VI materials, such as cadmium selenide (CdSe), can be synthesized from high temperature organometallic solution precursors.^1^ Ultraviolet (UV) and blue-emitting materials, such as zinc chalcogenides, present minimal environmental hazards. However, materials that emit across the visible spectral region (cadmium chalcogenides) and infrared (lead and mercury chalcogenides) are toxic or pose significant environmental risks–a problem if QDs are to be used in devices or biological and clinical applications. A significant research goal is the development of environmentally acceptable alternatives to cadmium, mercury and lead containing QDs, that emit strongly at the in the visible or infrared wavelengths.

A notable success is the development of indium phosphide (InP)-based QD materials, which are, in some circumstances, more efficient emitters than standard CdSe/ZnS core/shells QDs. InP/ZnS and their derivatives (such as InP/ZnSe/ZnS) can now routinely be prepared with near unity quantum yields (QYs), narrow emission profiles and in high yields.^2^ CuInE (E = S, Se) materials and related compounds are also prime candidates for green, heavy metal-free near-infrared (IR) emitters, with emission typically past 700 nm.^3−6^ The use of a II–VI material with a chalcopyrite semiconductor either as a heterostructure or core/shell material is well-known, notably in solar cell architectures. The lattice mismatch between chalcopyrite CuInS_2_ (*a* = 5.52 Å) and zinc blende ZnS (*a* = 5.41 Å) is low, at 2.0%, and similarly low for CuInS_2_ capped with zinc blended ZnSe (*a* = 5.67 Å) at 2.6%.^7−9^ and the preparation of such core/shell nanoparticles is relatively simple to realize.

In this report, we describe the development of Cu-based QDs with NIR emission, from synthesis to aqueous phase transfer. CuInZnS QDs were prepared and characterized before exploring core/shell and core/multishell structures, with both ZnSe and ZnS. The optical properties were optimized for NIR fluorescence imaging. To achieve phase transfer into water, the encapsulation of these materials within amphiphilic polymers was explored, providing high colloidal stability in aqueous media. The cytotoxicity of encapsulated QDs was tested and their potential use as an imaging agent is discussed. Finally, a Raspberry Pi-based imaging system was also developed for the easy and rapid imaging of NIR QD samples.

## Results and Discussion

Core QDs were synthesized with a hot-injection procedure using zinc diethyldithiocarbamate (Zn(DEDC)_2_), based on a previously reported method by Pons et al.,^3^ who used an asymmetric Zn dithiocarbamate complex to form of a reactive amine. We chose, however, to use the commercially available diethyldithiocarbamate complex based on its previous use in the synthesis of high quality nanomaterials.^10^ Observations made throughout the synthesis and characterization process indicated little difference between the particles prepared here using the symmetric dithiocarbamate and the asymmetric analogue discussed previously. The use of the Zn complex as a S source also resulted in the incorporation of Zn into the QD, which has been shown to be beneficial for the optical properties of CuInS_2_ QDs.^11−13^ The similar size of Zn and Cu cations allowed for Zn ions to fill Cu-vacancy sites that would otherwise result in nonradiative recombination. For convenience, the composition of these QDs is referred to as CuInZnS, although the actual stoichiometry is not 1:1:1:1. It is typical in the literature for Cu–In–S and Cu–In–Zn–S nanomaterials to be referred to as CuInS_2_ and CuInZnS (or ZnCuInS) for simplicity, despite reporting nonstoichiometric compositions. In this work, the elemental ratios of a range of QDs were found using X-ray photoelectron spectroscopy (XPS), energy dispersive X-ray spectroscopy (EDS) and inductively coupled plasma mass spectrometry (ICP-MS). Trioctylphosphine (TOP) and oleic acid (OA) were utilized as capping ligands, while oleylamine (OAm) was present primarily as an activating agent for the Zn(DEDC)_2_ precursor.

It has been shown previously that long chain primary amines can increase the reactivity of metal dithiocarbamates in order to reduce the decomposition temperature for QD synthesis.^14^ Hot-injection methods typically allow for better separation of nucleation and growth than “heat up” techniques, resulting in more monodisperse QD samples with greater uniformity in their properties and characteristics.^15^ Growth at 190 °C is rapid, with the appropriate QD size reached after 10 min. Upon hot injection of the dithiocarbamate complex, the color rapidly changes over the first 30 to 60 s from straw colored, through orange and red to black, indicating the progression to NIR-emitting QDs.

Transmission electron microscopy (TEM) of the sample after 10 min growth confirmed the formation of discrete 3.8 ± 0.3 nm (*n* = 40) particles, roughly spherical in shape and with well-defined lattice fringes (Figure S1a,b). Selected area electron diffraction (SAED, Figure S3) and X-ray diffraction (XRD) patterns both indicated crystalline nanoparticles with a cubic crystal structure. The (112), (204), and (312) planes of chalcopyrite were identified (Figure S1c,d) typical of CuInZnS and CuInS_2_ materials.^12,13,16^ A *d*-spacing of 0.32 nm was observed by TEM (Figure S1b) corresponding to the (112) plane of chalcopyrite CuInS_2_, further confirming the QDs structure. The broadness of the XRD peaks was consistent with small nanocrystalline particles, with an average size of 3.3 nm as estimated using the Scherrer equation.^17^ This value is close to that measured from TEM images, further confirming the formation of small crystalline nanoparticles.

X-ray photoelectron spectroscopy (XPS) was carried out to analyze the near surface composition of the CuInZnS QDs and Cu, In, Zn and S species were all found to be present (Figure S2). High resolution spectra of the Cu 2p region, revealed the characteristic spin–orbit split components, with the Cu 2p_3/2_ at a binding energy of 931.3 eV and consistent with Cu in the 1+ oxidation state in CuInS_2_ nanomaterials,^18^ no evidence of the 2+ oxidation state was found, which would be notable by a broader, higher energy shoulder to the Cu 2p peak and satellite structure at *ca.* 945 eV. The modified Auger parameter of 1848.7 eV was calculated from the Cu 2p_3/2_ binding energy and Cu L_3_M_45_M_45_ kinetic energy, close to the 1849.5 eV value reported for CuInS_2_ in the literature^19^ and the difference may be attributed to size effects. The In 3d_5/2_ and binding energy (444.4 eV) is again typical of In(III) species in CuInS_2_^18,20^ Similarly, the Zn 2p_3/2_ signal is found at 1020.5 eV, consistent with CuInZnS nanomaterials reported previously,^21^ while the S 2p_3/2_ binding energy of 161.4 eV is characteristic of sulfide in CuInS_2_ and CuInZnS nanomaterials.^18,20,21^ While not quantified, we do not discount the formation of a small amount of sulfate at the surface, exhibited by a small but broad around 169 eV, which form from the excess of the sulfur in the dithiocarbamate ligand. A stoichiometry of Cu_0.6_In_2.1_Zn_1.0_S_3.0_ was measured by quantitative analysis of the XPS derived atomic percentages.

Optical characterization of CuInZnS revealed a broad absorption band within the first minute of the reaction (Figure S3), typical of alloyed QDs, and an indistinct band edge absorption feature at 540 nm, typical of CuInS_2_ and CuInZnS.^11,16^ A corresponding photoluminescent (PL) peak was also observed at 700 nm with a full width at half-maximum (fwhm) of around 150 nm, and a Stokes shift of 160 nm for aliquots taken between 30 and 60 s. Both the absorption and PL features further progressed into the red and NIR, respectively, during the subsequent 9 min of growth. The change in emission during the reaction reflects the increased QD size and decreasing degree of exciton confinement, typical of QDs. The Stokes shift of 160 nm observed is atypical of QD band edge emission and indicates other radiative recombination pathways.^22^ Photoluminescence was observed for the final isolated core CuInZnS nanocrystals at 810 nm with a QY of 3.2 ± 0.2%, as measured using an integrating sphere. In comparison to CuInS_2_ nanocrystals in the literature, the QY is an order of magnitude greater due to the inclusion of Zn and its ability to fill defect Cu-vacancy sites.^11^

The emission wavelength could be controlled between 700 and 810 nm through variation of the experimental conditions, including temperature, growth time and Cu/In/Zn precursor ratios, (as demonstrated in Figure S4). Inductively coupled plasma mass spectrometry (ICP-MS) was used to quantify the amounts of Cu, In, and Zn in a range of samples with varying emission maxima. As previously noted, decreasing the amount of Cu relative to In results in red-shifted emission.^23^ ICP-MS also confirmed the incorporation of Zn from the Zn(DEDC)_2_ precursor which accounted for approximately 10% of the cation content in each nanocrystal sample, regardless of emission wavelength. Unfortunately, the concentration of S in the sample could not be obtained due to the overlap between ^16^O_2_ and ^32^S signals in ICP-MS. Energy dispersive X-ray spectroscopy was also used to quantify QD composition and a typical sample found to have a stoichiometry of Cu_2.0_In_2.7_Zn_1.0_S_5.5_. (1:1:1:4 precursor ratio) This suggests a higher incorporation of Zn than the ICP-MS measurements at 17.5% of the total cation content.

**Figure 1:**
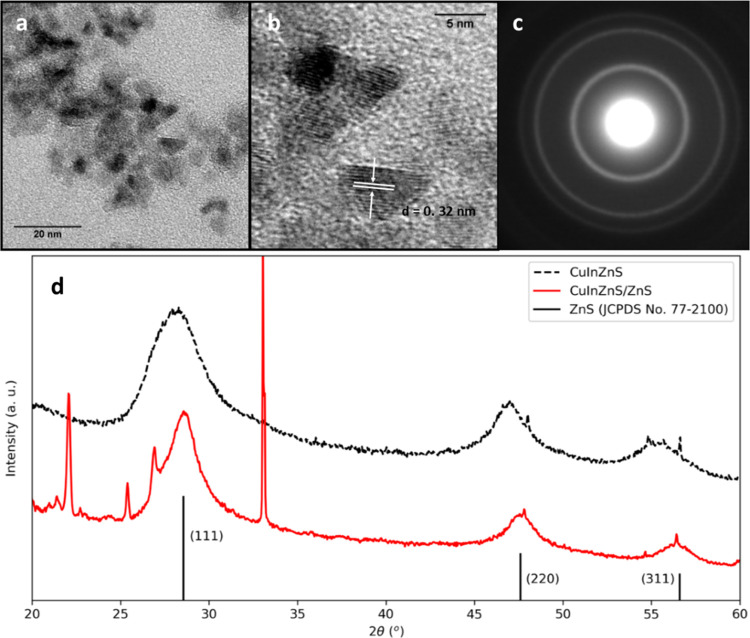
(a) TEM micrograph of CuInZnS/ZnS QDs and (b) magnification of lattice fringes (d-spacing = 0.32 nm). (c) SAED pattern indicating a cubic crystal structure, corresponding to (d) the XRD pattern showing a shift from the original CuInZnS position to higher angles.

The addition of a ZnS shell was used to improve the optical properties of CuInZnS QDs. Zn(DEDC)_2_ was further used as a single-source precursor for ZnS, with dropwise addition over 20 min at 230 °C. After ZnS shell growth, particle size had increased to 4.1 ± 0.5 nm (*n* = 40) as determined by TEM (Figure 1a,b), corresponding to 1.1 monolayers of ZnS (0.31 nm per monolayer).^24^ The SAED pattern (Figure 1c) again indicated a cubic crystal structure although a shift toward higher angles could be seen in the XRD pattern (Figure 1d), corresponding to the diffraction pattern of bulk ZnS. This highlights the presence of a ZnS shell on the Cu–In–Zn–S cores.

**Figure 2:**
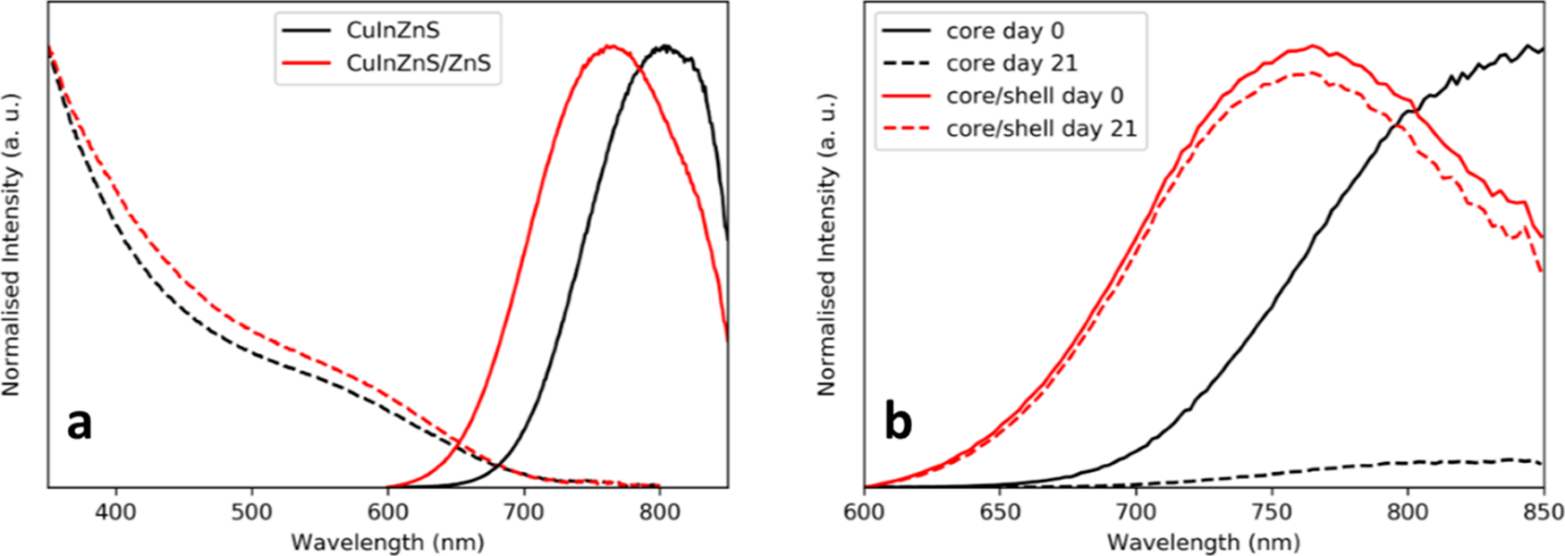
(a) Optical spectra of core CuInZnS (black) and core/shell CuInZnS/ZnS (red) QDs, with the absorption spectra plotted with dotted lines and the emission with solid lines. (b) The same samples over a 21-day period, normalized to the peak intensity at day zero. Excitation at 500 nm.

There was little change in the absorption characteristics of the material on shelling with ZnS; however, a 60 nm blue-shift of the PL emission from 810 to 750 nm was observed (Figure 2a). Diffusion of Zn ions into the core during high temperature shell growth likely resulted in a smaller effective core size and hence, shorter emission wavelength.^13^ Most importantly, on addition of a ZnS shell, the QY value increased from 3.2 ± 0.2 to 29 ± 3%, reflecting the passivation of defect sites in the core nanocrystal that could lead to nonradiative recombination of the exciton. Along with the improved QY, the photostability of the QDs was increased through the addition of ZnS shell. Figure 2b shows the change in relative fluorescence intensity for core and core/shell QDs over 21 days. Core fluorescence intensity dropped by 93.8%, while the intensity of the core/shell QDs only dropped by 6.2%.

The blue-shift in emission observed represents a significant problem for the development of precisely emitting fluorophores and is undesirable for biological imaging probes. Recent work by de Mello Donega and colleagues, however, addressed this issue and provides a method for minimal interfacial alloying.^25^ Evidence is put forward to suggest that a combination of high temperature and reactive precursors is required to maximize epitaxial growth and hence minimize blue-shift upon shelling.

Core/shell/shell CuInZnS/ZnSe/ZnS nanocrystals were synthesized, using modified procedures reportedly previously^26,27^ and as described in the Supporting Information. Justification by the authors for their use of an intermediary ZnSe shell is unclear and any advantages over CuInZnS/ZnS were not explained. Previously, intermediary shelling materials have been used to relieve the lattice strain which can occur between materials such as CdSe and ZnS.^28,29^ This can greatly improve the ease of epitaxial shell growth, allowing for thicker shells and improved optical properties. However, in this case, the lattice mismatch between CuInS_2_ and ZnS is already small at around 2%, meaning epitaxial shell growth occurs relatively unhindered. Figure S5 highlights the small difference in lattice parameter between CuInS_2_ and ZnS, as compared to CdSe and ZnS. Instead, we explored the inclusion of a ZnSe shell as a potential route to reducing the blue shift caused by diffusion of Zn into the CuInZnS core, observed when adding only a ZnS shell. This should be relatively straightforward, as the lattice mismatch on zinc blende ZnSe/ZnS is only 4.5%. It was hypothesized that the smaller band gap of ZnSe compared to ZnS could allow for greater wave function leakage, resulting in a red-shift of the PL. The use of a ZnSe layer has also been explored by Wang’s group, notably for shifting the optical gain in ZnSe_1–*x*_Te_*x*_ quantum dots.^30,31^

Growth of the ZnSe shell was achieved *via* a successive ion layer adsorption and reaction (SILAR) method in which the alternate addition of Zn and Se precursors resulted in the formation of one ZnSe monolayer at a time. This allowed for the controlled growth of a number of monolayers depending on the quantity of precursors used.^32^ Zn oleate was used as the Zn precursor, which was prepared *via* the reaction of ZnO and OA in 1-octadecene (ODE) at 250 °C. Elemental Se was used as the Se source and dissolved in tributyl phosphine (TBP). The SILAR addition was followed by an annealing step, in which the QD suspension was heated at 150 °C for 2 h to improve crystallinity and minimize the number of defects at the core/shell interface. The quantity of each reagent added during SILAR growth was calculated as described elsewhere.^33^ Addition of the outer ZnS shell was achieved in a similar method to the single shell QDs, using Zn(DEDC)_2_ as a single-source ZnS precursor.

**Figure 3:**
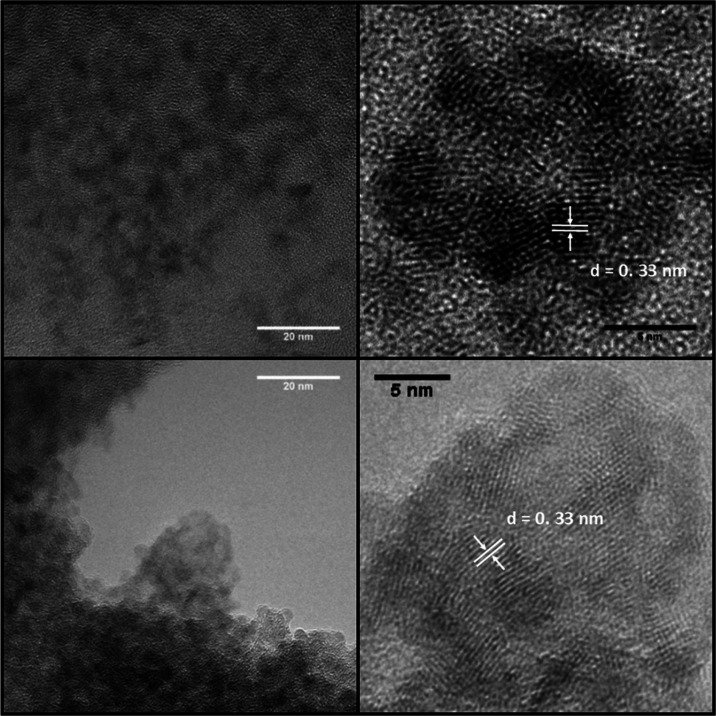
TEM images of CuInZnS/ZnSe (top row) and CuInZnS/ZnSe/ZnS (bottom row) QDs, with lattice spacings highlighted on the higher magnification images.

Core CuInZnS QDs with a diameter of 3.1 ± 0.4 nm (*n* = 25) were first coated with ZnSe to give CuInZnS/ZnSe nanocrystals of 3.5 ± 0.5 nm after annealing, indicating the addition of 1.2 monolayers (MLs) of ZnSe (for a calculated value of 0.33 nm per ML) (Figure 3). Prior to the annealing process, the QDs had an average diameter of 4.0 ± 1 nm (*n* = 40). The slight decrease in size and size distribution possibly occurs due to cation diffusion within the QD and loss of surface layers. Subsequent addition of a ZnS shell resulted in nanocrystals with a diameter of 4.1 ± 0.5 nm (*n* = 25), corresponding to the further addition of 2.1 MLs of ZnS (0.31 nm per ML).

No significant shifts or peak with changes were observed in the diffraction patterns upon addition of ZnSe and ZnS shells, as shown in Figure S6, and may indicate the formation of an alloyed surface rather than discrete shells.

**Figure 4:**
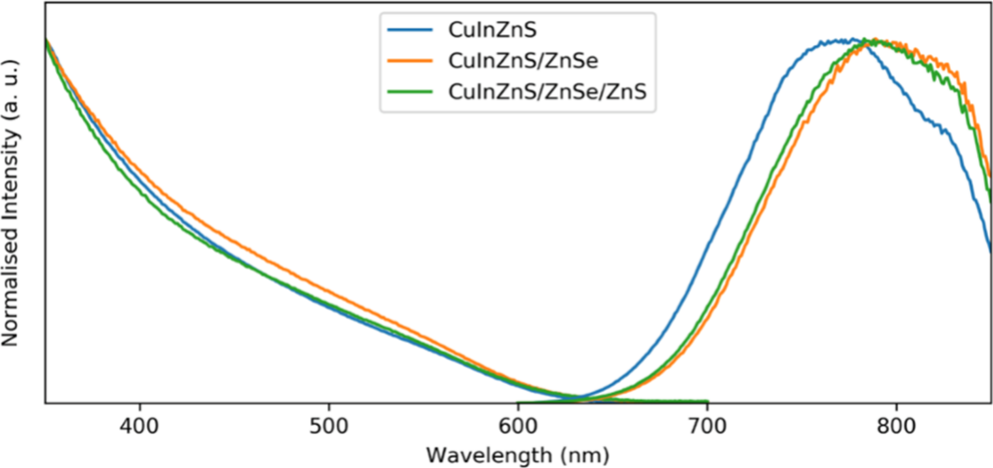
Absorption and PL spectra of core, core/shell and core/shell/shell QDs.

Figure 4 shows the 780 nm emission of the initial core QDs and the resulting red shift upon addition of a ZnSe shell to *ca.* 789 nm. In contrast to the addition of a ZnS shell discussed above, we suggest the lack of blue shift was due to limiting the diffusion of ions out of the core QDs, giving a minimal change in size and emission. The red shift to longer wavelength may reflect leakage of the exciton wave function from the core into the shell material, due to the smaller difference in band gap energies and hence less effective confinement.^34^Figure 4 shows no significant shift in the PL maximum during the annealing step although the emission profile narrowed, in keeping with the size focusing observed by TEM. Emission from CuInZnS/ZnSe/ZnS nanocrystals showed a slight 6 nm blue shift to 783 nm, which is most likely due to the large band gap ZnS shell aiding exciton confinement and not a change of size given the presence of the ZnSe shell. There was a 5 nm shift to lower wavelengths, a result of the annealing process, indicating a potential decrease in core size through ion diffusion although it is not clear why this does not occur during the first shelling procedure. The QY of the final core/shell/shell nanocrystals was measured absolutely to be 30.9 ± 0.4%, close to that of CuInZnS/ZnS QDs but at a slightly red-shifted wavelength more suited to NIR imaging.

In order to develop these for biological applications, phase transfer into water was required. Phase transfer can usually be achieved by either ligand exchange or encapsulation. Encapsulation retains the original organic surface ligands and, as a result, does not always result in such a large decrease in QY.^35^ Removal of the organic surface ligands can result in the formation of defect sites on the surface that, if not passivated, can allow for nonradiative recombination. Encapsulation is commonly used for the stabilization of QDs in water, although the resulting materials are often difficult to functionalize.^35^ Encapsulation of QDs and their surface ligands can, however, also result in particles with large hydrodynamic diameters, limiting their biological utility.

QD encapsulation was initially attempted through the use of hydrophobic, maleic anhydride (MA)-containing polymers as reported for iron oxide nanoparticles.^36,37^ In a typical reaction, poly(styrene maleic anhydride) (PSMA) or poly(maleic anhydride-*alt*-1-octadecene) (PMAO) was added to a suspension of QDs in chloroform and stirred for 1 h, followed by heating to 70 °C in a water bath and the dropwise addition of sodium hydroxide solution. To improve the encapsulation potential of the polymers, PMAO and PSMA were first functionalized with a hydrophilic, long chain amine as described elsewhere.^38,39^ The amine provided the hydrophilic moiety and are typically amine-functionalized poly(ethylene glycol) (PEG) chains, such as the Jeffamine. Amphiphilic polymers prepared in this way are favorable over phospholipids for QD encapsulation due to the significantly lower cost of the reagents needed.^35^ Not all the MA groups necessarily reacted to form amide linkages; the ratio of amine to MA groups was a significant variable in the success of QD encapsulation and is outlined in the experimental section. For both PMAO and PSMA, the number of maleic anhydride groups was estimated from the average molecular mass. In the case of PMAO, for example, 114 Jeffamine M1000 polymers were required on average to react with all the MA groups on a single PMAO polymer chain (average *M*_n_ = 30,000–50,000)

PMAO-Jeffamine and PSMA-Jeffamine polymers were added to QD suspensions in chloroform and stirred for 24 h to begin the encapsulation procedure. Subsequently the chloroform was removed under vacuum, leaving a brown film. Addition of potassium hydroxide solution and sonication for 30 s was then carried out to suspend the brown solid in water. In the case of PMAO-Jeffamine encapsulated QDs, the brown film could be suspended in 0.01 M KOH with sonication to give optically clear, brown dispersions of QDs in water. The same treatment with PSMA-Jeffamine QDs resulted in cloudy suspensions that were found to be unstable over a period of days, as shown in Figure S7.

Dynamic light scattering (DLS) analysis of the encapsulated QDs indicated that PSMA-Jeffamine resulted in the largest and most polydisperse structures, all at over a μm in diameter. Exact hydrodynamic diameters could not be determined due to the extreme polydispersity and are presumed to be above 1 μm regardless of QD capping agent. The inability of PSMA-Jeffamine to sufficiently stabilize QDs in water may be due to poor hydrophobic interactions between the phenyl group of styrene and the long chain capping ligands on the QD surface. We have included DLS intensity plots of the organic capped particles (TOP, OAm, and OA) and the polymer encapsulated particles to accompany the hydrodynamic diameter measurements in the Supporting Information (Figures S8 and S9, respectively).

**Figure 5:**
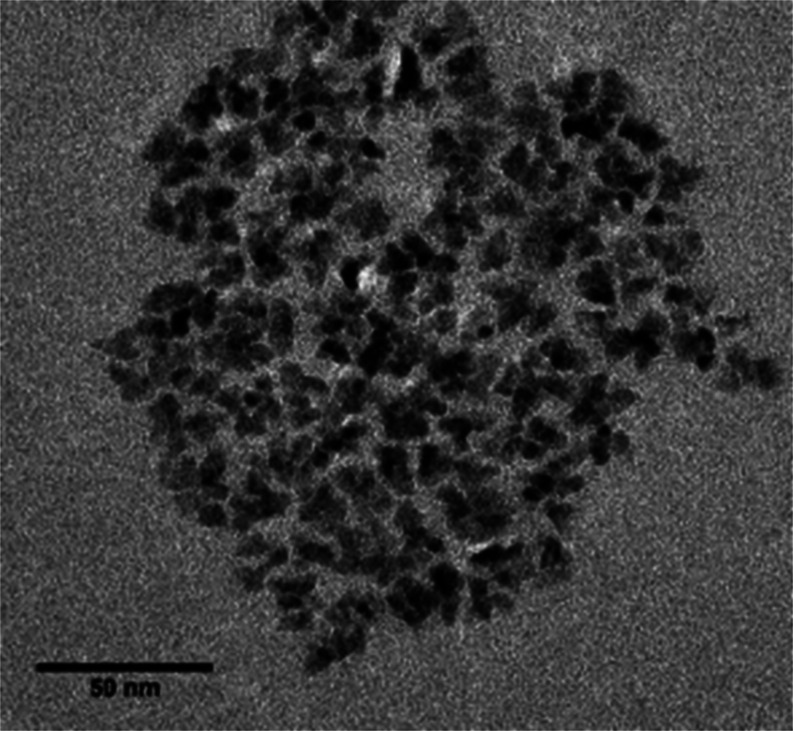
TEM image of an aggregate structure of PMAO-Jeffamine encapsulated, OA-capped QDs.

PMAO-Jeffamine encapsulated QDs, however, could be successfully characterized and diameters of 35 ± 11, 54 ± 18, and 350 ± 129 nm were measured for TOP-, OA- and OAm-coated QDs, respectively. The large size and polydispersity of the encapsulated OAm-capped QDs again matched the cloudy appearance of the aqueous suspension. This may reflect the difficulty in cleaning OAm-capped QDs, for which excess OAm can inhibit the formation of small polymer/QD micelles.^12^ While the resulting hydrodynamic size of these encapsulated QDs was significantly larger than the original QDs, the size was still within a relevant range for *in vivo* use. Particles up to around 50 nm can be successfully used for lymph node imaging, whereby QDs collect in the sentinel lymph nodes of a cancerous tissue due to the larger size.^40^ The success of the encapsulation with TOP- and OA-coated QDs was reflected in their emission properties (Figure S7) with brightest emission measured from QDs with the smallest hydrodynamic diameters. As expected, there was a decrease in QY from 30.9 ± 0.2 to 17 ± 5% (for OA-PMAO-capped QDs), most likely due to ligand stripping and aggregation. Of the phase transferred materials which exhibited bright emission after phase transfer (QD materials capped with either TOP or OA, and PMAO), the OA-capped QDs were of higher interest going forward as it expected that the cytotoxicity of OA is lower than that of TOP. Toxicity is an important concern in the encapsulation method as micelle components may be released *in vivo*, increasing the effective toxicity of the nanomaterial. Electron microscope images of the PMAO-Jeffamine encapsulated, OA-capped QDs indicated that while discrete QDs can still be seen, there are large aggregate structures present that may account for the large hydrodynamic diameters seen in the DLS measurements. Figure 5 shows one such structure, made up of over 100 individual QDs, indicating the encapsulation of multiple QDs.

Having established PMAO as the preferred polymer backbone over PSMA, the optimal polymer/amine ratio for QD encapsulation was verified. It has been reported that the polymer/Jeffamine ratio must be above *ca.* 1:80 to result in water-soluble polymer conjugates,^41^ corresponding to conjugation of amines to *ca.* 22% of the maleic anhydride groups. The remaining anhydride groups were opened with KOH to form carboxylic acid groups that aid in water solubility and electrostatic repulsion between polymers. In line with the reported observations, 1 and 5% polymer conjugates were not soluble at neutral pH or 0.01 M KOH. At 20 and 25% Jeffamine conjugation, the polymer could be dissolved in 0.01 M KOH, while higher percentages of amine allowed for the solubility in neutral pH water. The suspensions for 50% conjugation and greater were, however, initially cloudy due to the presence of chloroform that was not completely removed during preparation. A high degree of amine conjugation resulted in the formation of viscous mixtures as the chloroform was removed, inhibiting removal of the remaining solvent. These polymers were also not desirable given their very large molecular mass, particularly in the case of PMAO.

The PMAO-Jeffamine polymer conjugates with 20% amine and higher were used for the encapsulation of core/shell QDs to assess phase transfer efficacy. Both the 20 and 25% amine-modified polymers formed samples that could be resuspended in 0.01 M KOH to give optically clear solutions that retained NIR fluorescence. However, polymers with higher percentage Jeffamine conjugation (50, 75, and 100%) formed cloudy aqueous suspensions which proved to be unstable over time, with QDs and excess polymer precipitating out over a week. This is potentially due to the modified polymers being too large to adequately stabilize the QD, containing too many of the long chain hydrophilic groups.

As an assessment of significance of the ratio of QD to polymer, a 1:10 ratio by mass of QDs to PMAO-Jeffamine (25% amine conjugation) was compared to the 1:25 mass ratio used previously. The use of a lower polymer concentration was expected to lead to the formation of larger aggregate structures. However, it was observed that the 1:10 QD/polymer ratio resulted in an optically clear dispersion in water, with a hydrodynamic diameter of 40 ± 14 nm, as compared to 75 ± 18 nm for the 1:25 ratio. It was also found that the 20% conjugation of PMAO with Jeffamine resulted in smaller encapsulated QDs than the above-mentioned (higher) 25% modified-polymer, with the 1:10 ratio of QDs to PMAO-Jeffamine giving particles with a hydrodynamic diameter of 34 ± 11 nm, while the 1:25 ratio gave hydrodynamic diameters of 44 ± 18 nm. Most likely this is a direct result of the polymer with fewer Jeffamine chains being smaller and so forming smaller encapsulated particles. The small size of the lower QD/polymer ratio (1:10) may reflect a lower excess of polymer in solution after QD suspension in water.

Initial cytotoxicity assessment was carried out in HeLa cells using a microscope and visual assessment of cell health. The cells were incubated with QD concentrations up to 1 mg/mL over 48 h at 37 °C and observed under a light microscope. Up to 0.1 mg/mL, the majority of cells appear healthy with typical elongated shape and evidence of reproduction (Figure S8). This qualitative assessment of cytotoxicity allowed for continuation of testing using an MTT assay. Quantitative assessment of cytotoxicity was carried out in the murine macrophage J774.1 cell line, using an MTT assay. PMAO-Jeffamine encapsulated CuInZnS/ZnSe/ZnS QDs were found to have high compatibility up to 0.033 mg per mL although cell viability rapidly dropped above this concentration (Figure S9). Differences between the tolerance toward QDs of HeLa and J774 cells may explain why no detrimental effects were observed in HeLa cells by visual inspection. A control sample containing only the amphiphilic polymer ligand exhibited a cell viability of 100.5% at 0.1 mg/mL, a concentration higher than should be present in the most concentrated QD sample. The toxicity of these QDs at high concentration could be due either to the release of metal ions or organic capping agents into the cell medium and indicates poor suitability for *in vivo* imaging.

To rapidly assess the fluorescent properties of samples after synthesis, an inexpensive, simple NIR imaging system was developed using a Raspberry Pi computer. QDs that are fluorescent in the visible spectrum can be evaluated by eye using illumination from a UV lamp or laser pointer, but this is not possible for the dark brown QD suspensions that emit in the NIR. The initial outcome of a reaction cannot easily be ascertained until full optical characterization is carried out. A simple imaging system was assembled using a Raspberry Pi computer and Raspberry Pi “NoIR” camera module, along with a 715 nm long-pass filter to block out visible light. The sensitivity of Si-based CMOS sensors in consumer products are optimized for detection in the visible region although the absorption of Si extends up to around 1100 nm, corresponding to the 1.12 eV band gap.^42^ Typically, NIR filters are used to block wavelengths above 650 nm although the “NoIR” camera module has this filter removed.

**Figure 6:**
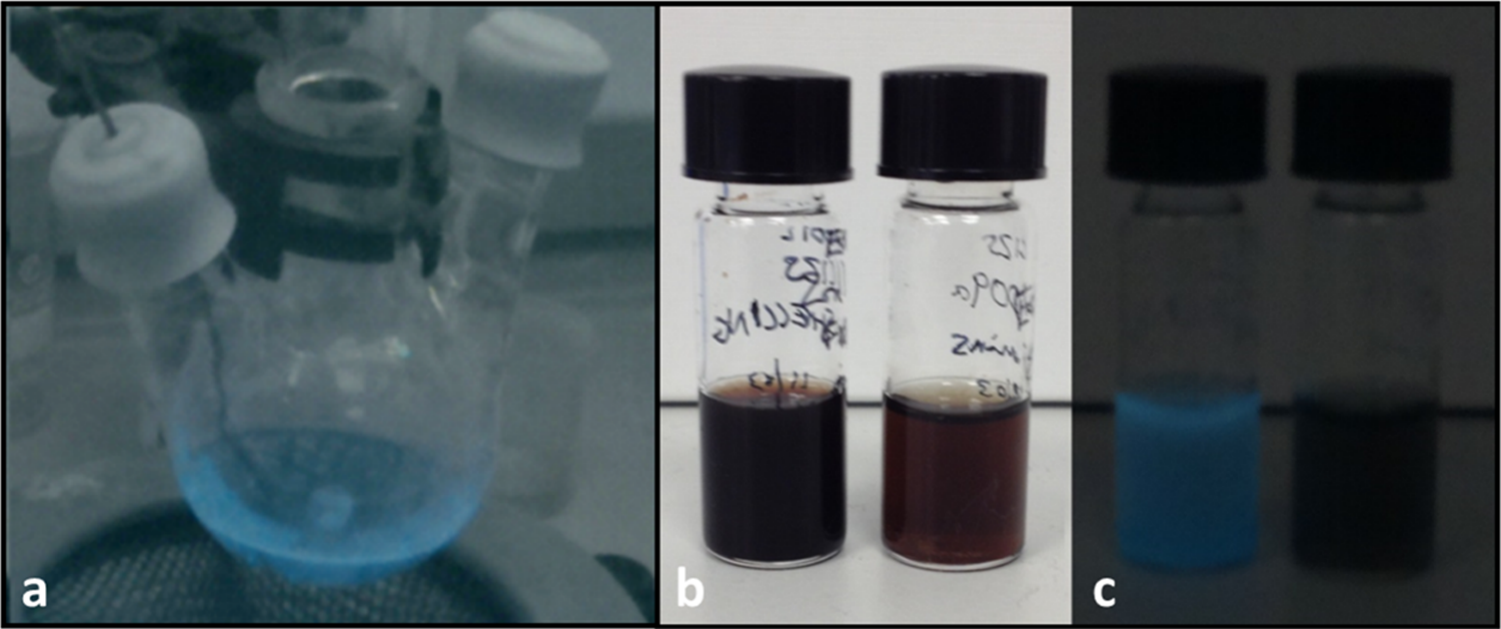
(a) NIR image of CuInZnS QDs directly after synthesis and (b) comparison of a luminescent and nonluminescent sample by eye. (c) The same samples as observed by a smart phone and Raspberry Pi “NoIR” camera under excitation by room lights in ambient conditions.

Figure 6a demonstrates the use of the camera to assess the NIR PL of a sample after synthesis, and prior to processing. It should be noted that fluorescence cannot be detected during synthesis due to the decrease in QY at higher temperatures as demonstrated for CuInZnS QDs elsewhere.^43^ Image 6b shows two samples of colloidal CuInZnS NCs, of which one is NIR fluorescent, and one is not. To the naked eye, the samples are indistinguishable, and their emission properties are not obvious without performing a spectroscopic measurement. Figure 6c shows the same samples as viewed using the NIR camera, where the sample on the left can be seen to fluoresce under the ambient of lighting of the lab.

**Figure 7:**
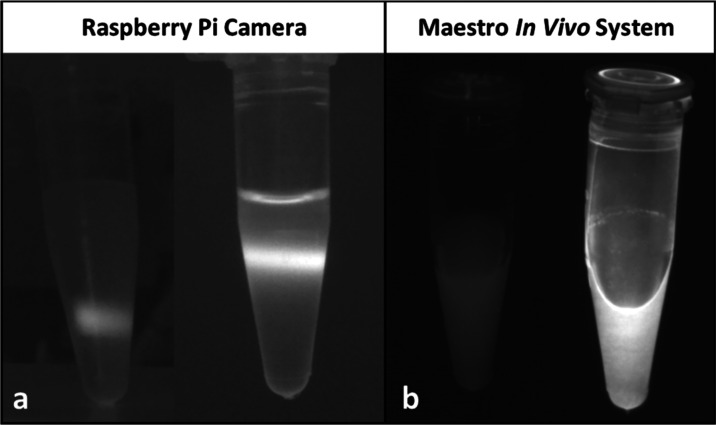
NIR images of core (left) and core/shell (right) QDs taken with (a) a Raspberry Pi NIR camera (samples excited using a 450 nm laser pointer) and (b) a commercial *in vivo* imaging system from Maestro (455 nm excitation).

The broad absorption of the Cu-based QDs across the visible spectrum means that ambient light from overhead fluorescent lighting was sufficient to excite the QDs. For samples with poor QY, typically below 5%, samples could be excited with a laser pointer of various wavelengths (450, 532, or 650 nm) to ensure sufficient PL for detection by the camera. This is demonstrated in Figure 7, in which the output of the Raspberry Pi camera is compared to that from a commercially available imaging system (Maestro). The NIR camera also allowed for quick assessment of the progress of the phase transfer reactions discussed above. Crucially, the camera shows whether the QY has decreased below a usable quantity.

A recent publication has demonstrated the use of a similar, simple system in thyroidectomies for identification of the parathyroid glands.^44^ Only autofluorescence of the glands in the NIR was detected but this approach could be extended to the detection of NIR-emitting fluorophores currently used in procedures such as lymph node mapping and tumor margin identification.^45^ While widespread clinical use of such a system is unlikely, the utility of a NIR camera in the development of fluorescent materials in a laboratory setting is evident–particularly because many standard spectrometers or optical microscopes do not have detectors that reach into the NIR or IR ranges.

## Conclusions

In conclusion, CuInZnS/ZnSe/ZnS nanocrystals have been synthesized with optical properties suitable for a NIR fluorescence imaging probe. Importantly, NIR emission could be achieved while maintaining a small overall nanocrystal size that favors more rapid biological clearance *in vivo*. The core/shell/shell species exhibited similar optical properties to those of the core/shell nanocrystals through interface engineering, with both similar QY and emission wavelength values. To achieve water-soluble QDs, an encapsulation route was explored that used conjugates of PMAO and Jeffamine M1000. Core/shell/shell QDs were successfully transferred into water *via* this method, while retaining a high quantum yield, a hydrodynamic diameter below 50 nm, and exhibiting negligible toxicity at low concentrations.

## Supporting Information Available

Experimental section, CuInS_2_ synthesis, CuInZnS/ZnS synthesis, CuInZnS/ZnSe/ZnS synthesis, successive ion layer adsorption and reaction calculations, nanocrystal cleaning, preparation of amine-modified amphiphilic polymers, encapsulation of CuInZnS/ZnS quantum dots, J477A.1 cell culturing and MTT cytotoxicity assay, HeLa cell incubation and live/dead staining, optical characterization, X-ray diffraction pattern analysis, transmission electron microscopy, X-ray photoelectron spectroscopy, dynamic light scattering, inductively coupled plasma mass spectrometry, near-infrared imaging, supporting references (PDF)

## References

[ref1] Murray C. B.; Norris D. J.; Bawendi M. G. Synthesis and characterization of nearly monodispersed CdE (E = sulfur, selenium, tellurium) semiconductor nanocrystallites. J. Am. Chem. Soc. 1993, 115, 8706–8715. 10.1021/ja00072a025.

[ref2] Gazis T. A.; Cartildge A. J.; Matthews P. D. Colloidal III-V quantum dots: a synthetic perspective. J. Mater. Chem. C 2023, 11, 3926–3935. 10.1039/D2TC05234B.

[ref3] Pons T.; Pic E.; Lequeux N.; Casette E.; Bezdetnaya L.; Guillemin F.; Marchal F.; Dubertret B. Cadmium-free CuInS_2_/ZnS quantum dots for sentinel lymph node imaging with reduced toxicity. ACS Nano 2010, 4, 2531–2538. 10.1021/nn901421v. 20387796

[ref4] Roy P.; Mukherjee A.; Mondal P.; Bhattacharyya B.; Narayan A.; Pandey A. Electronic structure and spectroscopy of I-III-VI2 nanocrystals: A perspective. J. Phys. Chem. C 2022, 126, 7364–7373. 10.1021/acs.jpcc.1c10922.

[ref5] Jain S.; Bharti S.; Bhullar G. K.; Tripathi S. K. I-III-VI core/shell QDs: Synthesis, characterizations and applications. J. Lumin. 2020, 219, 116912 10.1016/j.jlumin.2019.116912.

[ref6] Regulacio M. D.; Han M.-Y. Multinary I-III-VI_2_ and I_2_-II-IV-VI_4_ semiconductor nanostructures for photocatalytic applications. Acc. Chem. Res. 2016, 49, 511–519. 10.1021/acs.accounts.5b00535. 26864703

[ref7] Wei S.-H.; Zunger A. Band offsets and optical bowing of chalcopyrites and Zn-based II-VI alloys. J. Appl. Phys. 1995, 78, 3846–3856. 10.1063/1.359901.

[ref8] Chang J.-Y.; Su L.-F.; Li C.-H.; Chang C-. C.; Lin J.-M. Efficient ″green″ quantum dot-sensitized solar cells based on Cu_2_S-CuInS_2_-ZnSe architecture. Chem. Commun. 2012, 48, 4848–4850. 10.1039/c2cc31229h. 22498756

[ref9] Hofmann A.; Pettenkofer C. Surface orientation dependant band alignment for CuInSe_2_-ZnSe-ZnO. Appl. Phys. Lett. 2011, 98, 113503 10.1063/1.3567758.

[ref10] Shen S.; Zhang Y.; Peng L.; Xu B.; Du Y.; Deng M.; Xu H.; Wang Q. Generalized synthesis of metal sulfide nanocrystals from single-source precursors: Size, shape and chemical composition control and their properties. CrystEngComm 2011, 13, 4572–4579. 10.1039/c0ce00982b.

[ref11] Nakamura H.; Kato W.; Uehara M.; Nose K.; Omata T.; Otsuka-Yao-Matsuo S.; Miyazaki M.; Maeda H. Tunable photoluminescence wavelength of chalcopyrite CuInS_2_-based semiconductor nanocrystals synthesized in a colloidal system. Chem. Mater. 2006, 18, 3330–3335. 10.1021/cm0518022.

[ref12] Li L.; Daou T. J.; Texier I.; Chi T. T. K.; Liem N. Q.; Reiss P. Highly Luminescent CuInS_2_/ZnS Core/Shell Nanocrystals: Cadmium-Free Quantum Dots for In Vivo Imaging. Chem. Mater. 2009, 21, 2422–2429. 10.1021/cm900103b.

[ref13] Park J.; Kim S.-W. CuInS_2_/ZnS core/shell quantum dots by cation exchange and their blue-shifted photoluminescence. J. Mater. Chem. 2011, 21, 3745–3750. 10.1039/c0jm03194a.

[ref14] Jung Y. K.; Kim J. I.; Lee J. Thermal Decomposition Mechanism of Single- Molecule Precursors Forming Metal Sulfide Nanoparticles. J. Am. Chem. Soc. 2010, 132, 178–184. 10.1021/ja905353a. 20000670

[ref15] Yarema M.; Yarema O.; Lin W. M. M.; Volk S.; Yazdani N.; Bozygit D.; Wood V. Upscaling Colloidal Nanocrystal Hot-Injection Syntheses via Reactor Underpressure. Chem. Mater. 2017, 29, 796–803. 10.1021/acs.chemmater.6b04789.

[ref16] Chen C.-W.; Wu D.-Y.; Chan Y.-C.; Lin C. C.; Chung P.-H.; Hsiao M.; Liu R.-S. Evaluations of the Chemical Stability and Cytotoxicity of CuInS_2_ and CuInS_2_/ZnS Core/Shell Quantum Dots. J. Phys. Chem. C 2015, 119, 2852–2860. 10.1021/jp510908f.

[ref17] Langford J. I.; Wilson A. J. C. Scherrer after sixty years: A survey and some new results in the determination of crystallite size. J. Appl. Crystallogr. 1978, 11, 102–113. 10.1107/S0021889878012844.

[ref18] Pan D.; An L.; Sun Z.; Hou W.; Yang Y.; Yang Z.; Lu Y. Synthesis of Cu-In-S Ternary Nanocrystals with Tunable Structure and Composition. J. Am. Chem. Soc. 2008, 130, 5620–5621. 10.1021/ja711027j. 18396869

[ref19] Biesinger M. C. Advanced analysis of copper X-ray photoelectron spectra. Surf. Interface Anal. 2017, 49, 1325–1334. 10.1002/sia.6239.

[ref20] Lee J.; Han C.-S. Large-scale synthesis of highly emissive and photostable CuInS_2_/ZnS nanocrystals through hybrid flow reactor. Nanoscale Res. Lett. 2014, 9, 78 10.1186/1556-276X-9-78. 24533662 PMC3996094

[ref21] Guo W.; Chen N.; Tu Y.; Dong C.; Zhang B.; Hu C.; Chang J. Synthesis of Zn-Cu-In-S/ZnS Core/Shell quantum dots with inhibited blue-shift photoluminescence and applications for tumor targeted bioimaging. Theranostics 2013, 3, 99–108. 10.7150/thno.5361. 23422883 PMC3575590

[ref22] Fuhr A.; Yun H. J.; Makarov N. S.; Li H.; McDaniel H.; Klimov V. I. Light-Emission Mechanism in CuInS_2_ Quantum Dots Evaluated by Spectral Electrochemistry. ACS Photonics 2017, 4, 2425–2435. 10.1021/acsphotonics.7b00560.

[ref23] Liu L.; Hu R.; Law W.-C.; Roy I.; Zhu J.; Ye L.; Hu S.; Zhang X.; Yong K.-T. Optimizing the synthesis of red- and near-infrared CuInS_2_ and AgInS_2_ semiconductor nanocrystals for bioimaging. Analyst 2013, 138, 6144–6153. 10.1039/c3an01030a. 23967444

[ref24] Wu P.; Fang Z.; Zhong X.; Yang Y. J. Depositing ZnS shell around ZnSe core nanocrystals in aqueous media via direct thermal treatment. Colloids Surf., A 2011, 375, 109–116. 10.1016/j.colsurfa.2010.11.070.

[ref25] Berends A. C.; van der Stam W.; Hofman J. P.; Bladt E.; Meeldijk J. D.; Bals S.; de Mello Donega C. Interplay between Surface Chemistry, Precursor Reactivity, and Temperature Determines Outcome of ZnS Shelling Reactions on CuInS_2_ Nanocrystals. Chem. Mater. 2018, 30, 2400–2413. 10.1021/acs.chemmater.8b00477. 29657360 PMC5895981

[ref26] Tan Z.; Zhang Y.; Xie C.; Su H.; Liu J.; Zhang C.; Dellas N.; Mohney S. E.; Wang Y.; Wang J.; Xu J. Near-band-edge electroluminescence from heavy-metal-free colloidal quantum dots. Adv. Mater. 2011, 23, 3553–3558. 10.1002/adma.201100719. 21732559

[ref27] Liu W.; Zhang Y.; Ruan C.; Wang D.; Zhang T.; Feng Y.; Gao W.; Yin J.; Wang Y.; Riley A. P.; Hu M. Z.; Yu W. W. ZnCuInS/ZnSe/ZnS Quantum Dot-Based Downconversion Light-Emitting Diodes and Their Thermal Effect. J. Nanomater. 2015, 2015, 298614 10.1155/2015/298614.

[ref28] Reiss P.; Carayon S.; Bleuse J.; Pron A. Low polydispersity core/shell nanocrystals of CdSe/ZnSe and CdSe/ZnSe/ZnS type: Preparation and optical studies. Synth. Met. 2003, 139, 649–652. 10.1016/S0379-6779(03)00335-7.

[ref29] Talapin D. V.; Mekis I.; Götzinger S.; Kornowski A.; Benson O.; Weller H. CdSe/CdS/ZnS and CdSe/ZnSe/ZnS core-shell-shell nanocrystals. J. Phys. Chem. B 2004, 108, 18826–18831. 10.1021/jp046481g.

[ref30] Huang Z.; Sun Q.; Wang S.; Shen H.; Cai W.; Wang Y. Broadband tunable optical gain from ecofriendly semiconductor quantum dots with near-half-exciton threshold. Nano Lett. 2023, 23, 4032–4038. 10.1021/acs.nanolett.3c00813. 37125767

[ref31] Wu Y.; Cai W.; Huang Z.; Ren Y.; Wu Y.; Wang Y. An all-colloidal and eco-friendly quantum-dot laser. Laser Photonics Rev. 2024, 18, 2301167 10.1002/lpor.202301167.

[ref32] Chang J.; Waclawik E. R. Colloidal semiconductor nanocrystals: controlled synthesis and surface chemistry in organic media. RSC Adv. 2014, 4, 23505–23527. 10.1039/C4RA02684E.

[ref33] Li J. J.; Wang Y. A.; Guo W.; Keay J. C.; Mishima T. D.; Johnson M. B.; Peng X. Large-Scale Synthesis of Nearly Monodisperse CdSe/CdS Core/Shell Nanocrystals Using Air-Stable Reagents via Successive Ion Layer Adsorption and Reaction. J. Am. Chem. Soc. 2003, 125, 12567–12575. 10.1021/ja0363563. 14531702

[ref34] Dabbousi B. O.; Rodriguez-Viejo J.; Mikulec F. V.; Heine J. R.; Mattoussi H.; Ober R.; Jensen K. F.; Bawendi M. G. (CdSe)ZnS Core - Shell Quantum Dots : Synthesis and Characterization of a Size Series of Highly Luminescent Nanocrystallites. J. Phys. Chem. B 1997, 101, 9463–9475. 10.1021/jp971091y.

[ref35] Tyrakowski C. M.; Snee P. T. A primer on the synthesis, water-solubilization, and functionalization of quantum dots, their use as biological sensing agents, and present status. Phys. Chem. Chem. Phys. 2014, 16, 837–855. 10.1039/C3CP53502A. 24296551

[ref36] Lasheras X.; Insausti M.; de Muro I. G.; Garaio E.; Plazaola F.; Moros M.; De Matteis L.; de la Fuente J. M.; Lezama L. Chemical Synthesis and Magnetic Properties of Monodisperse Nickel Ferrite Nanoparticles for Biomedical Applications. J. Phys. Chem. C 2016, 120, 3492–3500. 10.1021/acs.jpcc.5b10216.

[ref37] Moros M.; Ambrosone A.; Stepien G.; Fabozzi F.; Marchesano V.; Castaldi A.; Tino A.; de la Fuente J. M.; Tortiglione C. Deciphering intracellular events triggered by mild magnetic hyperthermia in vitro and in vivo. Nanomedicine 2015, 10, 2167–2183. 10.2217/nnm.15.70. 25959578

[ref38] Lees E. E.; Nguyen T. L.; Clayton A. H. A.; Mulvaney P. The Preparation of colloidally stable, water-soluble, biocompatible, semiconductor nanocrystals with a small hydrodynamic diameter. ACS Nano 2009, 3, 1121–1128. 10.1021/nn900144n. 19388661

[ref39] Speranskaya E. S.; Sevrin C.; De Saeger S.; Hens Z.; Goryacheva I. Y.; Grandfils C. Synthesis of Hydrophilic CuInS_2_/ZnS Quantum Dots with Different Polymeric Shells and Study of Their Cytotoxicity and Hemocompatibility. ACS Appl. Mater. Interfaces 2016, 8, 7613–7622. 10.1021/acsami.5b11258. 26963807

[ref40] Li J.; Jiang B.; et al. Advances and perspectives in nanoprobes for noninvasive lymph node mapping. Nanomedicine 2015, 10, 1019–1036. 10.2217/nnm.14.201. 25867863

[ref41] Speranskaya E. S.; Beloglazova N. V.; Lenain P.; De Saeger S.; Wang Z.; Zhang S.; Hens Z.; Knopp D.; Niessner R.; Potapkin D. V.; Goryacheva I. Y. Polymer-coated fluorescent CdSe-based quantum dots for application in immunoassay. Biosens. Bioelectron. 2014, 53, 225–231. 10.1016/j.bios.2013.09.045. 24140873

[ref42] Gouveia L. C. P.; Choubey B. Advances on CMOS image sensors. Sens. Rev. 2016, 36, 231–239. 10.1108/SR-11-2015-0189.

[ref43] Liu W.; Zhang Y.; Zhai W.; Wang Y.; Zhang T.; Gu P.; Chu H.; Zhang H.; Cui T.; Wang Y.; Zhao J.; Yu W. W. Temperature-dependent photoluminescence of ZnCuInS/ZnSe/ZnS quantum dots. J. Phys. Chem. C 2013, 117, 19288–19294. 10.1021/jp4024603.

[ref44] Kim Y.; Kim S. W.; Lee K. D.; Ahn Y.-C. Real-time localization of the parathyroid gland in surgical field using Raspberry Pi during thyroidectomy: a preliminary report. Biomed. Opt. Express 2018, 9, 3391–3398. 10.1364/BOE.9.003391. 29984104 PMC6033547

[ref45] Landau M. J.; Gould D. J.; Patel K. M. Advances in fluorescent-image guided surgery. Ann. Transl. Med. 2016, 4, 392 10.21037/atm.2016.10.70. 27867944 PMC5107411

