# Supplementary material for: Interface Engineering of Water-Dispersible Near-Infrared-Emitting CuInZnS/ZnSe/ZnS Quantum Dots
Source: Cryst Growth Des. 2024 Jul 18;24(15):6275–83. doi: 10.1021/acs.cgd.4c00528 (PMC11311135; doi:10.1021/acs.cgd.4c00528)
Supplement: Supplementary file 1 — cg4c00528_si_001.pdf [file cg4c00528_si_001.pdf]

SUPPORTING INFORMATION FOR

Title: Interface engineering of water-dispersible near-infrared-emitting CuInZnS/ZnSe/ZnS quantum dots.

Authors: Patrick Mann,[1] Simon M. Fairclough,[1] Struan Bourke,[1] Mary Burkitt Gray,[1] Laura Urbano,[2] David J. Morgan,[3] Lea Ann Dailey,[4] Maya Thanou,[5] Nicholas J. Long,[6] Mark A. Green.[1]*

Addresses:

1) Department of Physics, King's College London, The Strand, London, WC2R 2LS, UK.
2) Centre for Topical Drug Delivery and Toxicology, School of Life and medical Sciences, University of Hertfordshire, Hatfield, AL10 9AB, UK.
3) School of Chemistry, Cardiff University, Main Building, Park Place, Cardiff, CF10 3AT, UK.
4) Department of Pharmaceutical Sciences, University of Vienna, Josef-Holaubek-Platz 2, 1090 Vienna, Austria.
5) Institute of Pharmaceutical Science, King's College London, 150 Stamford Street, London, SE1 9NH, UK.
6) Department of Chemistry, Imperial College London, Molecular Sciences Research Hub, White City Campus, W12 0BZ, UK.

Table of Contents

Experimental section *pg. 2*
$CuInS_2$ synthesis *pg. 2*
CuInZnS/ZnS Synthesis *pg. 3*
CuInZnS/ZnSe/ZnS Synthesis *pg. 3*
Successive Ion Layer Adsorption and Reaction Calculations *pg. 3*
Nanocrystal Cleaning *pg. 4*
Preparation of Amine-Modified, Amphiphilic Polymers *pg. 5*
Encapsulation of CuInZnS/ZnS Quantum Dots *pg. 5*
J477A.1 Cell Culturing and MTT Cytotoxicity Assay *pg. 6*
HeLa Cell Incubation and Live/Dead Staining *pg. 7*
Optical Characterisation *pg. 7*
X-Ray Diffraction Pattern Analysis *pg. 8*
Transmission Electron Microscopy *pg. 8*
X-Ray Photoelectron Spectroscopy *pg. 9*
Dynamic Light Scattering *pg. 9*
Inductively Coupled Plasma Mass Spectrometry *pg. 9*
Near Infrared Imaging *pg. 10*
Figure S1 *pg. 10*
Figure S2 *pg. 11*
Figure S3 *pg. 12*
Figure S4 *pg. 12*
Figure S5 *pg. 13*
Figure S6 *pg. 13*
Figure S7 *pg. 14*
Figure S8 *pg. 15*

Figure S9 *pg. 15*
References *pg. 16*

Experimental section

Copper(I) chloride (CuCl, 99.99%), indium(III) chloride ($InCl_3$, 99.995%) and zinc nitrate hexahydrate ($Zn(NO_3)_2{\cdot}6H_2O$, 98%) were supplied by Acros Organics. Sulfur powder (100 mesh, sublimed), selenium powder (100 mesh, 99.99%), tributylphosphine (TBP, 97%), trioctylphosphine (TOP, 97%), zinc diethyldithiocarbamate, (ZDEC, $[Zn(S_2CNEt_2)_2]$, 98%), zinc oxide (ZnO, 99%), 1-octadecene (ODE, 90%, technical grade), oleic acid (OA, 90%, technical grade), oleylamine (OAm, 70%, technical grade), methylenediphosphonic acid (MDP), zinc stearate, tetrahydrofuran (THF, 99% with BHT as inhibitor), poly(maleic anhydride-*alt*-1-octadecene) (PMAO, average Mn = 30,000-50,000 g mol-1), poly(styrene maleic anhydride) (PSMA, cumene-terminated, average Mn = 1,600 g mol-1) and nitric acid (70%) were obtained from Sigma Aldrich. Hexane (HPLC grade) and toluene (HPLC grade) were supplied by Fisher. Jeffamine M1000 was supplied by Huntsman. Ultrapure water (Mili-Q System) was used throughout, and all materials used as received unless stated otherwise. Nanocrystal synthesis carried out under a nitrogen atmosphere, making use of glove box and Schlenk line techniques.

J774A.1 cell line was acquired from American Type Culture Collection (ATCC®, Virginia, USA). HeLa cells were supplied by Klaus Suhling within the Department of Physics at King's College London.

Purified HPLC water (impurities ≤1ppb and anions ≤ 0.1 mg/Kg, cat # 34877-2.5L); NaCl, Triton® X-100 (4-(1,1,3,3-Tetramethylbutyl), Trypan blue solution (cat # T8154), Hemoglobin standard (human, cat # H7379-1g), 3-(4,5-dimethylthiazol-2-yl)-2,5-diphenyl tetrazolium blue (MTT) were purchasedfrom Sigma Aldrich (UK). High glucose DMEMphenol red (cat # 32430027) and phenol red free (cat # 31053028) CCM, Pen Strep (10,000 units of penicillin and 10 mg streptomycin/mL); L-glutamine 200 mM; HEPES buffer 1M, pyruvate 100 mM, heat inactivated FBS 10500 (cat # 10500-064), 96-well black plate (Greiner bio-one, cat# 655090) were obtained from Gibco® (Life TechnologiesTM, Paisley, UK). Dulbecco's Modified Eagle Medium (DMEM), foetal bovine serum (FBS), penicillin–streptomycin-L-glutamine solution, and trypsin/EDTA solution (TE) were purchased from Thermofisher Scientific. Eight well microplates were obtained from Thistle Scientific Ltd. Ultrapure water (Direct-Q3 system, Millipore) used throughout unless otherwise stated. Materials also used as received unless otherwise stated.

CuInZnS Synthesis

CuCl (19.8 mg, 0.2 mmol) and $InCl_3$ (44.2 mg, 0.2 mmol) were dissolved in a mixture of TOP (2 mL), OlAm (2 mL) and ODE (10 mL). The solution was degassed under vacuum at 70^oC for 30 minutes. Under a nitrogen atmosphere, the reaction was heated to 190^oC before the rapid injection of

$[Zn(S_2CNEt_2)_2]$ (72.4 mg, 0.2 mmol) and Zn stearate (380 mg, 0.6 mmol) in TOP (1 mL). The temperature was maintained at 190°C for 10 minutes before allowing the reaction mixture to cool to room temperature. QDs were collected and processed as described below (Section 0).

CuInZnS/ZnS Synthesis

An aliquot of previously synthesised CuInZnS nanocrystals in hexane and TOP (2 mL) were added to ODE (10 mL) and OA (2 mL). The solution was then degassed under vacuum at 70°C for 30 minutes. The solution was heated to 230°C and a mixture of $[Zn(S_2CNEt_2)_2]$ (72.4 mg, 0.2 mmol) and Zn stearate (379 mg, 0.6 mmol) in ODE (8 mL), OlAm (1 mL) and TOP (1 mL), injected dropwise over 20 minutes. The solution was cooled to room temperature and QDs collected as described below.

CuInZnS/ZnSe/ZnS Synthesis

To prepare the precursor solutions, ZnO (100.3 mg) was added to OA (2.073 g) and ODE (10 mL) before heating to around 250°C under N_2. The high temperature was maintained until a clear, pale yellow solution of zinc oleate was obtained. Separately, $[Zn(S_2CNEt_2)_2]$ (181.0 mg) was added to TBP (6 mL) and ODE (24 mL), before heating to 70°C, giving a clear and colourless solution. Tributylphosphine was used instead of trioctylphosphine (TOP) whilst referring to Bowen Katari *et al.* who highlighted that the ligand would be removed preferentially in the high temperature environment over TOP [1].

The as-synthesised solution of core CuInZnS nanocrystals in ODE, described above, was heated to 200°C before the addition of 2 mL Se/TBP solution followed by 2 mL zinc oleate solution, both dropwise. After 10 minutes a further 2 mL of Se/TBP and 2 mL of zinc oleate solution were added dropwise, before reducing the temperature to 150°C for 2 hours. This was followed by the dropwise addition of 3 mL $[Zn(S_2CNEt_2)_2]$ solution at 200°C. The solution was again reduced to 150 °C for 2 hours of annealing before cooling to room temperature. QDs were collected and processed as described below.

Successive Ion Layer Adsorption and Reaction Calculations

The concentrations used in the ZnSe shell growth reaction above were calculated based on methods described in the literature [2], [3]. In summary, the volume of a single core QD was first calculated using the radius measured by TEM and converted to a mass using the density of the bulk material. The theoretical mass of QDs produced in the reaction was then calculated using the expected number of moles and molecular weight. Using these two calculated values, the number of moles of particles could be calculated.

For the subsequent calculation of shell precursors, several shell material parameters was obtained, including the lattice parameter, a, the crystal structure (unit cell structure) and molecular volume (also known as the "crystallographic density"), V_{shell}. The molecular volume of a crystalline solid is related to the number of formula units, Z, and the volume of the unit cell, V_{cell}, which in turn is calculated from the lattice parameter using Equation 1, assuming a cubic system.

$$V_{cell} = a^3 (1)$$

Calculating Z for the unit cell of a given material involved working out the mass of a single unit cell from V_{cell} and the material density. Converting to several moles using the molar mass and multiplying by Avogadro's constant then gave the number of formula units per unit cell.

$$V_{shell} = \frac{V_{cell}}{Z} (2)$$

Next, the thickness, d, and volume of a single monolayer (ML) of shell material was calculated using Equations 3 and 4.

$$d_{ML} \frac{a^3}{\sqrt{3}} (3)$$

$$V_{ML}(n) = \frac{4}{3}((r_{core} + (n \times d_{ML}))^3 - r_{core}^3 (4)$$

Finally, knowing the volume of a single ML or shell material and the crystallographic density, the number of molecular units and hence the number of moles of precursor required was calculated.

$$Number\ of\ molecular\ units\ per\ ML = \frac{V_{ML}(1)}{V_{shell}} (5)$$

For the calculation of the SILAR growth of ZnSe, it was assumed the cores were spherical QDs with a composition of CuInZnS and a radius of 1.9 nm as measured by TEM. For simplicity, the bulk density of 4.73 g cm^{-1} for $CuInS_2$ was also used. A lattice parameter and bulk density of 0.567 nm and 5.26 g cm^{-1} respectively were used for ZnSe ML thickness and volume calculations.

Nanocrystal Cleaning

Crude nanocrystal dispersions were diluted with hexane and MeOH, approximately doubling the volume of liquid, before centrifugation at 1000 rpm for 5 minutes. The lower, clear MeOH layer was removed, and the upper, brown organic layer was processed further. Nanocrystals were precipitated through the addition of excess MeOH and centrifugation at 4000 rpm for 5 minutes. The precipitated

particles could be resuspended in hexane, toluene or ODE as appropriate. The initial excess ligand removal step may be repeated as necessary to achieve sufficient cleaning for TEM and XRD analysis.

Preparation of Amine-Modified, Amphiphilic Polymers

A solution of Jeffamine M1000 (0.94 mmoles) in CHCl3 (15 mL) was added dropwise to dry polymer (0.012 mmoles, PMAO or PSMA) with vigorous stirring. The polymer solution was left stirring overnight and used without any further purification.

Encapsulation of CuInZnS/ZnS Quantum Dots

Three samples of CuInZnS/ZnSe/ZnS QDs were first capped with either TOP, OA or OAm *via* an organic-phase ligand exchange detailed in the literature [4]. Cleaned QDs were suspended in neat ligand (TOP, OA or OAm) and stirred for 24 hours. The QDs were precipitated with IPA and recovered *via* centrifugation at 5000 rpm for 5 minutes before repeating the ligand exchange process twice more.

In a typical ligand encapsulation procedure, amine-modified polymer in $CHCl_3$ (3.125 μmol) was added to a QD solution (0.5 mL, 10 mg mL) to give a QD to polymer mass ratio of 1:25 (quantities adjusted as required for other ratios). These solutions were left to mix overnight. To transfer the particles into water, aqueous KOH (3 mL, 0.01 mol dm^{-3}) was added and the mixture sonicated for 2 minutes. The suspended QDs in water were filtered through a 0.45 μm filter (hydrophilic polyamide, Satorius) and washed twice with water in a centrifuge filter (Vivapsin, 10 kDa MWCO) with centrifugation at 4000 rpm for 10 minutes.

The molar ratios of Jeffamine to PMAO used, along with the resulting percentage of Jeffamine conjugation.

**Percentage of MA groups reacted[a] (%)**	**Molar ratio of amine to PSMA**	**Molar ratio of amine to PMAO**
1	1:0.1	1:1
5	1:0.5	1:6
20	1:1.8	1:23
25	1:2.3	1:29
50	1:4.5	1:57
75	1:6.8	1:86

100	1:9.0	1:114

J477A.1 Cell Culturing and MTT Cytotoxicity Assay

J774A.1 cells were cultured in DMEM supplemented with 10% FBS, 1% antibiotics (100 U penicillin and 0.1 mg/ml streptomycin), 5% L-glutamine (200 mM), 1% sodium pyruvate (100 mM), and 1% HEPES 1 M. The cells were grown in complete DMEM medium with phenol red, in a humidified atmosphere at 5% CO_2 and at 37°C. Subconfluent cultures were subcultured by scraping and dispensed into new flask 1-9 x 10^5 cells mL^{-1}. Prior to MTT Assay, wells A-2 to H-11 of a 96-well plate 8000 cells/well were seeded (200 µL of complete medium were added to the remaining cells) remaining wells and the plate was incubated in a humidified atmosphere at 5% CO_2 and at 37°C for 24 hours to allow cells to attach to the plate.

The tetrazolium salt MTT colorimetric assay was used to assess the J774A.1 cell line survival following incubation with QDs. The assay was performed with minor modifications from the protocol, originally described by Tim Mosmann in 1983 and is based on the principle that NAD(P)H-dependent cellular oxidoreductase enzymes may, under defined conditions, reflect the number of viable cells present [209]. The mitochondrial dehydrogenase enzymes cleave the MTT tetrazolium salt (3-(4,5-dimethylthiazol-2-yl)-2,5-diphenyltetrazolium bromide), forming its insoluble formazan. The formazan is impermeable to the cell membranes and accumulates in healthy cells; after the reaction the formazan crystals are dissolved and quantified by measuring the absorbance which is proportional to the cell viability.

QD suspensions (0.1-300 µg/mL) in phenol red free DMEM were prepared from a 1.5 mg mL^{-1} stock. The medium covering cells after 24h attachment was replaced with 200 µL of QD suspension at different concentrations and controls of water dilution. Medium only was added to negative control wells (no treatment) and to positive control wells (for later addition of Triton X-100 solution). As dilution control, complete DMEM diluted with sterile water at the same proportion as the nanoparticles at the highest concentration (300 µg/mL) was prepared (ratios of 1:4 water in medium). After dosing, plates were incubated in a humidified atmosphere at 5% CO_2 and at 37°C for 24 hours. Following incubation, the medium in positive control wells was replaced by 100 µL of Triton-X 1% solution and the plates were incubated for 10 minutes in a humidified atmosphere at 5% CO_2 and at 37°C. Cells were then washed twice with 200 µL of PBS at 37°C, and then covered with 1 mg mL^{-1} MTT solution and incubated overnight. After incubation, 100 µL of a solution composed of 10% SDS and 50% DMF were added to each well in order to solubilise the formazan crystals and incubated for 24 hours at 37°C. Subsequently, the absorbance of the plate was read at λ = 570 nm with background subtraction at 650

nm in a plate reader Spectramax 340 PC384 (Program Softmax pro 6.2.2). The percentage of cell viability was calculated according to the equation below:

$$Viability = \frac{A_S - A_P}{A_N - A_P} \times (6)$$

where, A_S is the absorbance of the treated cells, A_P is the absorbance of the positive control and A_N is the absorbance of the negative control.

HeLa Cell Incubation and Live/Dead Staining

HeLa cells were cultured as separate adherent monolayers in DMEM supplemented with 10% heat inactivated FBS and 1% penicillin–streptomycin-L-glutamine solution. The cells were washed with PBS, then treated with TE to detach the HeLa cells to seed to further culture plates. HeLa cells were incubated (37°C under 5% CO_2 in humidified air) on a sterilised 8 square well microplate at a cell density of 0.8×10^6 upon reaching confluency for 24 hours. Cleaned NCs suspended in water were diluted in DMEM to give a range of concentrations. 150 μL of NC suspension was added to 150 μL of the aforementioned media in an 8 well plate. These were incubated for a further 24 hours. HeLa cells were fixed in a 4% formaldehyde solution for 15 minutes, and then washed with phosphate-buffered saline (pH 7.0) six times.

For live/dead staining, the cells were incubated (37°C, 5% CO_2) with NC or control samples (free GSH ligand) for 24 hours, before washing with PBS and incubating with Nuc488 for 15 minutes at room temperature in the absence of light. The cells were washed twice with PBS at room temperature and fixed with 4% paraformaldehyde (in PBS) for 20 min at room temperature. Cells were permeabilized by incubation (37°C, 5% CO_2) with 0.3% Triton X-100 in blocking buffer (0.5% bovine serum albumin, 0.1% NaN_3 in PBS) for 5 minutes followed by 3 x 5 minute incubations (37°C, 5% CO_2) in blocking buffer and 20 mM glycine. Fixed cells were imaged on an inverted Nikon Eclipse microscope using wide-field epifluorescence (Ti-E) equipped with a Cool SNAP HQ 2, DS-Fi2 Color CCD camera and 20x air objective. Original data were processed using NIS elements software (Nikon) and ImageJ (version no. 1.51j8 http://imagej.nih.gov/ij/, 1997–2018). The number of stained (dead) cells and total number of cells were counted manually in ImageJ. Cell viability was reported as the percentage of unstained cells ± one standard deviation (n = 3). Cell culturing and staining was performed with the help of Struan Bourke at King's College London.

Instrumentation

Optical Characterisation

Absorption spectra were acquired on a U4100 (Hitachi) UV-visible-NIR spectrophotometer, with sample and reference solvent in quartz cuvettes (1 cm pathlength). Photoluminescence (PL) spectra were recorded on a Fluoromax 4 (Horiba) fluorometer, with samples in quartz cuvettes. Cleaned nanocrystal samples were suspended in an appropriate solvent, typically hexane or water.

Quantum yield measurements were made both via an absolute method using an integrating sphere and by dye comparison as indicated in the main text. Absolute measurements were taken using a Quantaurus C11347 (Hamamatsu) PL QY spectrometer. Cleaned QD samples were suspended in hexane or water as appropriate and measured in the supplied proprietary quartz cuvettes (1 cm pathlength). Prior to measuring the QY, the optical density of each sample was verified as 0.1 or below. The dye comparison method was used as outlined elsewhere in the literature [5]. In short, two samples were prepared with an optical density at the excitation wavelength (typically 500 nm) of 0.1 or below and the fluorescence spectrum of each recorded as above. The fluorescence peak was integrated and used along with the optical density values and solvent refractive index in the following equation,

$$QY_{NP} = QY_{ref}\frac{I_{NP}}{I_{ref}}\frac{A_{ref}}{A_{NP}}\frac{n_{NP}^2}{n_{ref}^2} (7)$$

where *ref* indicates the reference dye, *NP* indicates the nanoparticulate sample, *I* is the area under the fluorescence curve, *A* is the absorption at a given wavelength and *n* is the refractive index of the solvents used.

X-Ray Diffraction Pattern Analysis

Powder X-ray diffraction (XRD) analysis was carried out in the Department of Materials at Imperial College London with the help of Mr Richard Sweeney. Thoroughly cleaned nanocrystal samples were prepared as high concentration suspensions in hexane and deposited onto silicon wafers (2.5 cm^2) prior to measurement on an MPD X-ray diffractometer (PANalytical) with a Cu K-α (λ = 0.1541 nm) X-ray source. The Scherrer equation (below) was used to approximate the mean nanocrystal size from the full width at half maximum (FWHM) of the largest peak [6].

$$D = \frac{0.9\lambda}{dcos\theta} (8)$$

Here, D is the mean nanocrystal size in nanometres, λ is the X-ray wavelength in nanometres, d is the FWHM in radians and θ is the scattering angle of the diffraction peak.

Transmission Electron Microscopy

Transmission electron microscopy (TEM) was carried out on either an FEI Tecnai T20 or Jeol 2100 microscope (acceleration voltages of 200 kV) for standard resolution imaging and selected area electron diffraction (SAED). Energy-dispersive X-ray spectroscopy (EDS) measurements were recorded on an Oxford Instruments detector. Thoroughly cleaned QD samples were deposited from toluene or water as appropriate on ultrathin carbon film supported, 3 mm gold grids (400 mesh, TAAB). Particle size and size distribution values were estimated from TEM images and given as the average (± one standard deviation) diameter for 30 particles unless otherwise stated. TEM micrographs and SAED patterns were analysed with ImageJ software (version no. 1.51j8 http://imagej.nih. gov/ij/, 1997–2019).

X-Ray Photoelectron Spectroscopy

A Kratos Axis Ultra DLD system was used to collect X-ray photoelectron spectroscopy (XPS) spectra using monochromatic Al Kα X-ray source operating at 150 W (10 mA x 15 kV), courtesy of David Morgan at Cardiff University for HarwellXPS. Data was collected with pass energies of 80 eV for survey spectra, and 40 eV for the high-resolution scans with step sizes of 1 eV and 0.1 eV respectively. The system was operated in the Hybrid mode, using a combination of magnetic immersion and electrostatic lenses and acquired over an area approximately 300 x 700 μm^2. A magnetically confined charge compensation system was used to minimize charging of the sample surface, and all spectra were taken with a 90° take off angle. A base pressure of ~ $1x10^{-9}$ Torr was maintained during collection of the spectra. Transmission corrected data was analysed using CasaXPS (v2.3.19rev1.1l) after subtraction of a Shirley background and using modified Wagner sensitivity factors as supplied by the instrument manufacturer.

Dynamic Light Scattering

Hydrodynamic diameter and ζ-potential measurements were obtained by dynamic light scattering (DLS) using a Zetasizer Nano-S (Malvern). All samples were recorded at 25°C in water using quartz cuvettes (1 cm pathlength) for size measurements or in disposable ζ-potential cuvettes (Malvern) for surface charge measurements. In all cases, the refractive index of $CuInS_2$ (RI = 2.51) and an absorption value of 0.1 was used.

Inductively Coupled Plasma Mass Spectrometry

Composition analysis by inductively coupled plasma mass spectrometry (ICP-MS) was carried out on a NexION 350D (Perkin Elmer) with the help of Andy Cakebread at the King's College London mass spectrometry facility. QD samples were thoroughly cleaned, digested in concentrated nitric acid (1 mL,

70%) and diluted with water to give a final element concentration of 2000 ppb or less and a nitric acid concentration of 7%. Each QD sample was prepared in triplicate and the concentrations averaged.

Near Infrared Imaging

Raspberry Pi NIR imaging was carried out using a Raspberry Pi "NoIR" camera module (#2357308, Farnell UK) and Raspberry Pi 2 (model B, #2461030, Farnell UK), in conjunction with a 715 nm long pass coloured glass filter (FGL715, Thorlabs UK). Sample excitation was achieved with ambient light in the laboratory. NIR images of CuInZnS/ZnS QDs in hexane were also acquired using the Maestro EX imaging system (Perkin-Elmer), with excitation at 661 nm and detection at 770 nm.

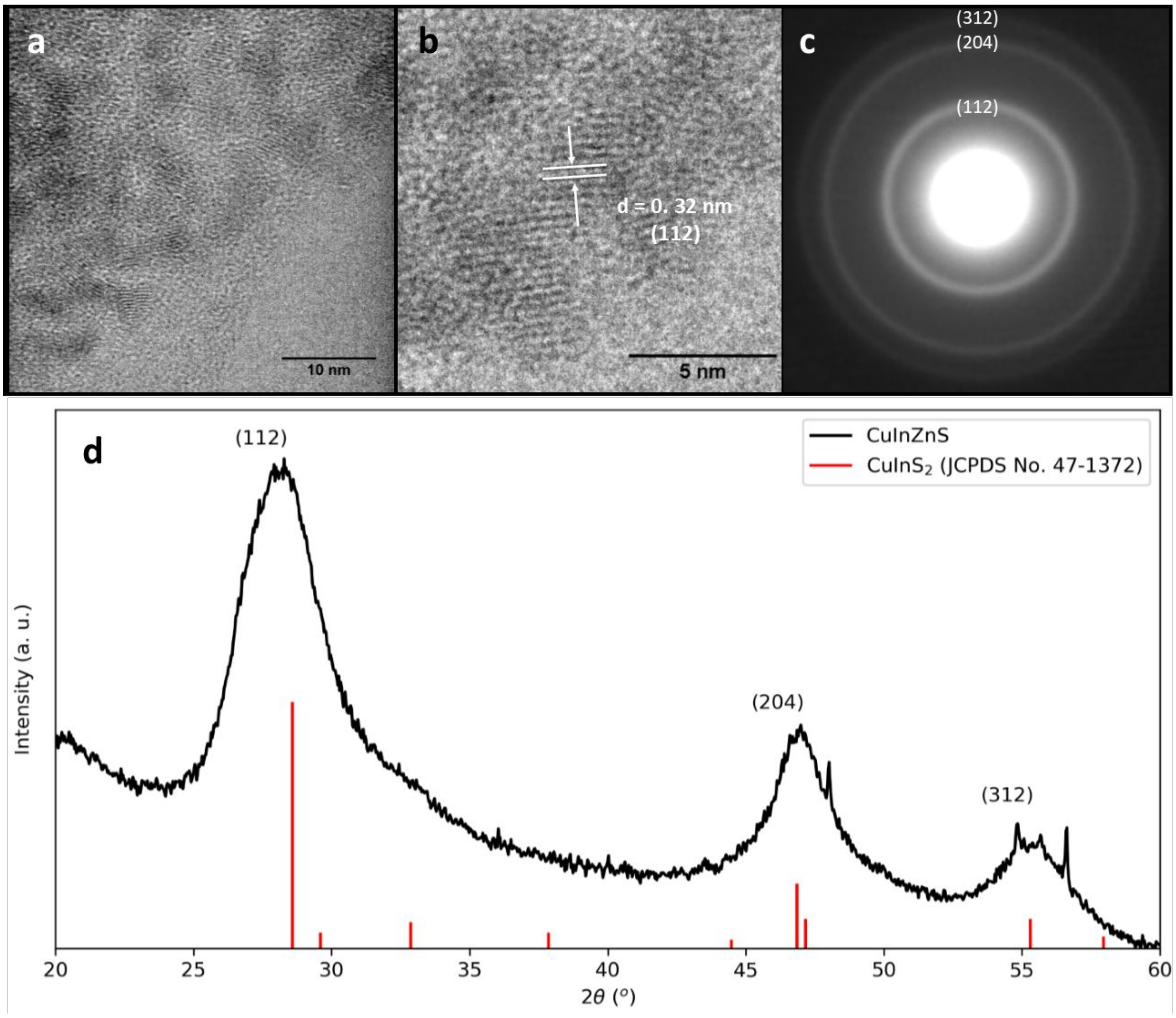

Figure S1. a) TEM micrograph of CuInZnS QDs. b) Magnified image of CuInZnS lattice fringes. c) SAED pattern indicating a cubic crystal structure. d) XRD pattern of CuInZnS QDs, matching the

chalcopyrite crystal phase of $CuInS_2$ (JCPDS No. 47-1372). Sharp peaks can be attributed to the Si substrate used.

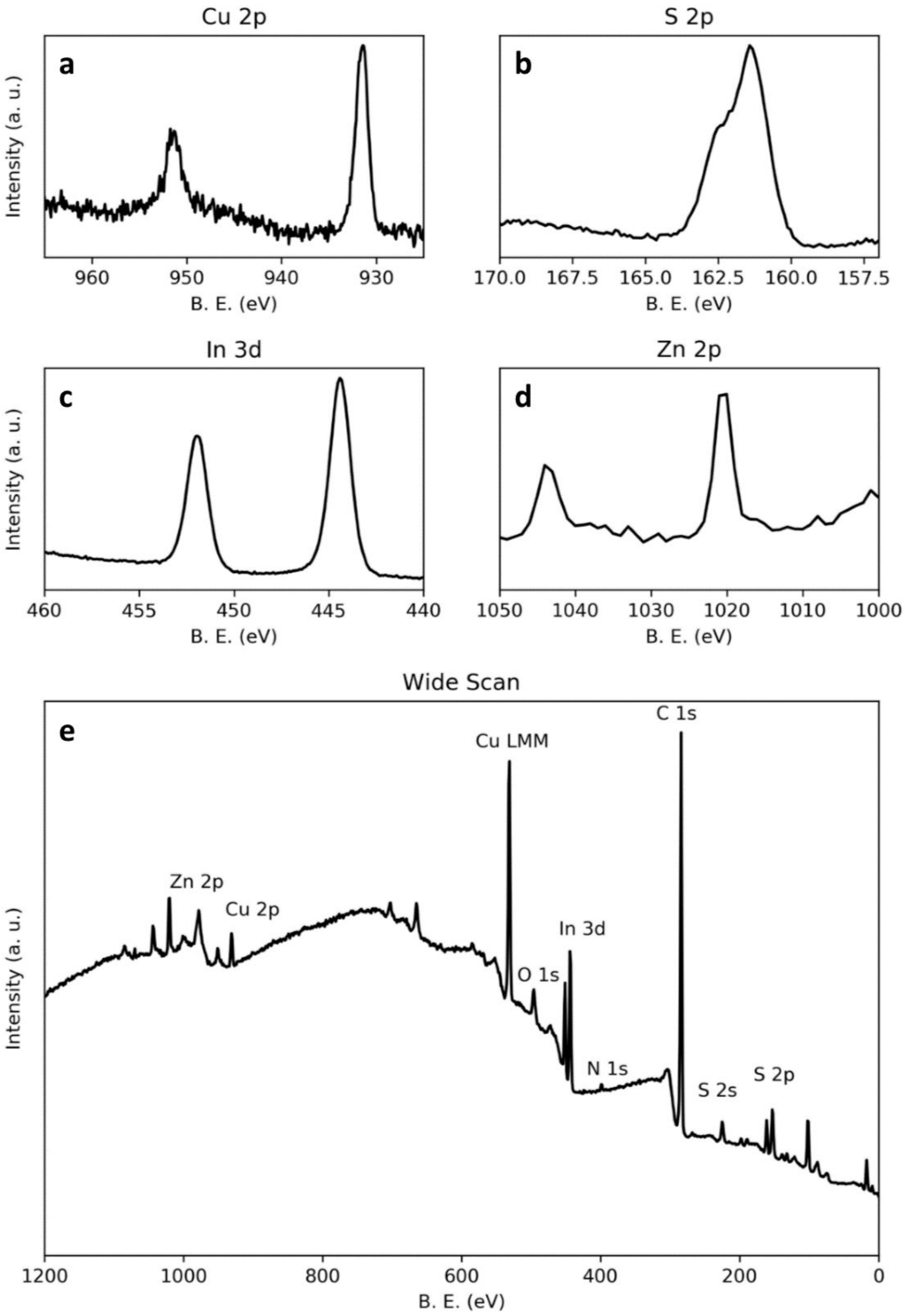

Figure S2. XPS spectra of CuInZnS QDs. High resolution scans were recorded for a) Cu 2p, b) S 2p, c) In 3d and d) Zn 2p, along with e) a survey scan from 0 to 1200 eV.

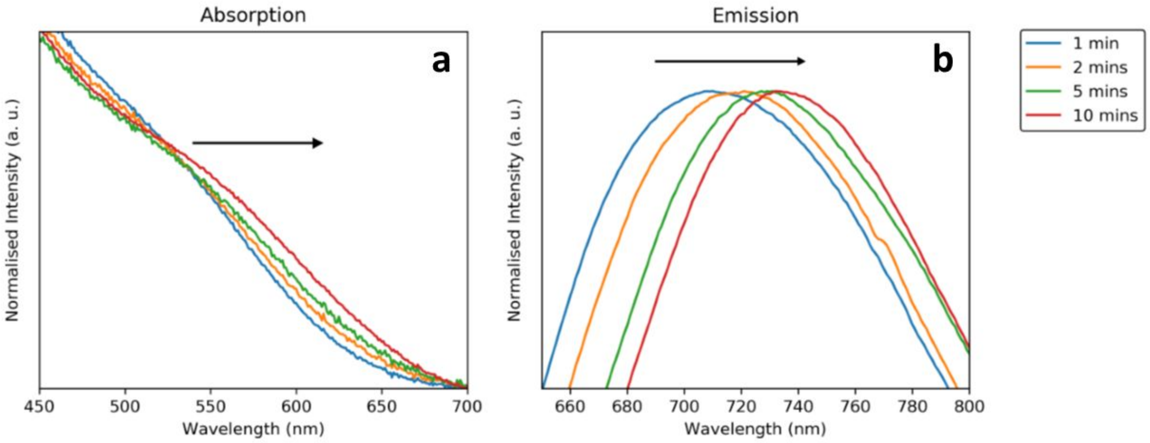

Figure S3. a) Absorption and b) photoluminescence spectra of aliquots taken during the hot-injection synthesis of CuInZnS QDs. Excitation at 500 nm.

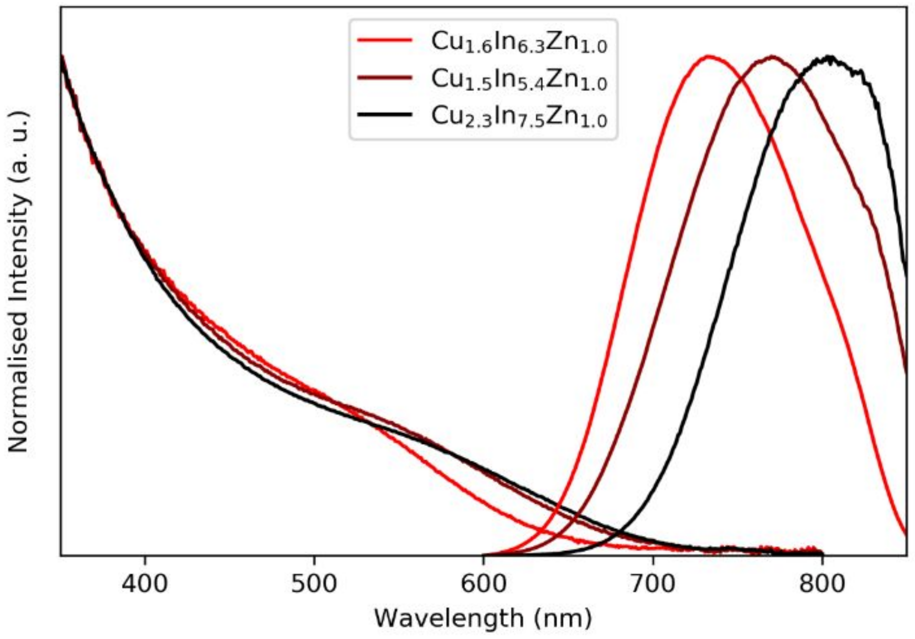

Figure S4. Absorption and photoluminescence spectra for core CuInZnS QDs with varying Cu:In:Zn ratios. Excitation at 500 nm.

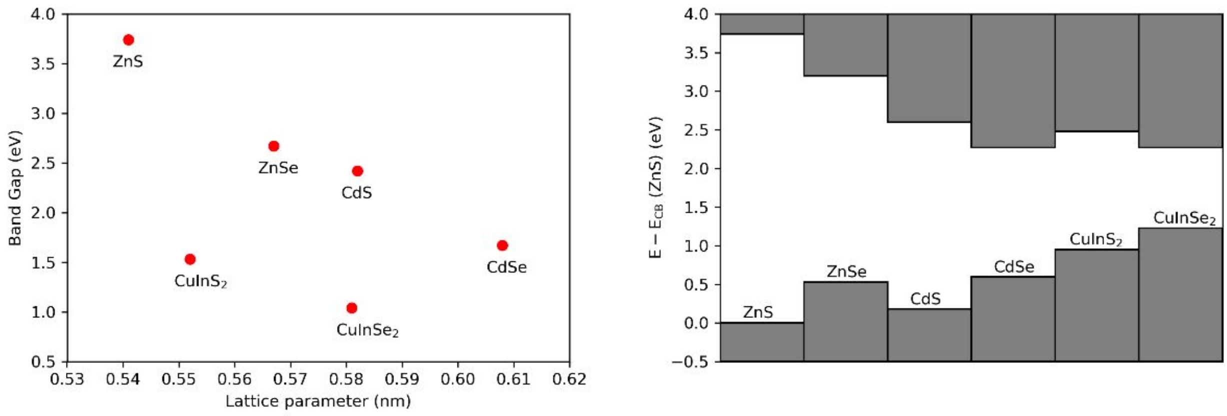

Figure S5. Left: a plot of band gap and lattice parameter for a range of I-III-VI and II-VI semiconductors. Right: band alignments for the same semiconductors with each band gap relative to that of ZnS.

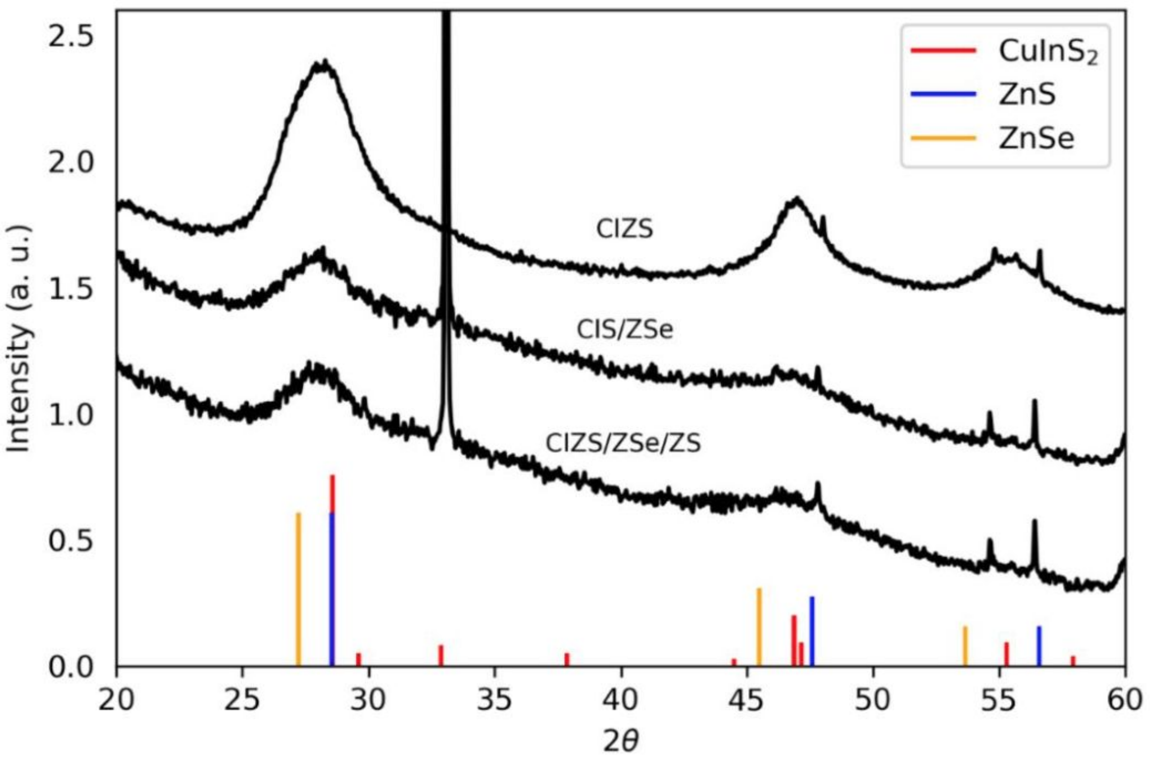

Figure S6. XRD patterns of core, core/shell and core/shell/shell QDs. Sharp peaks can be attributed to the Si substrate.

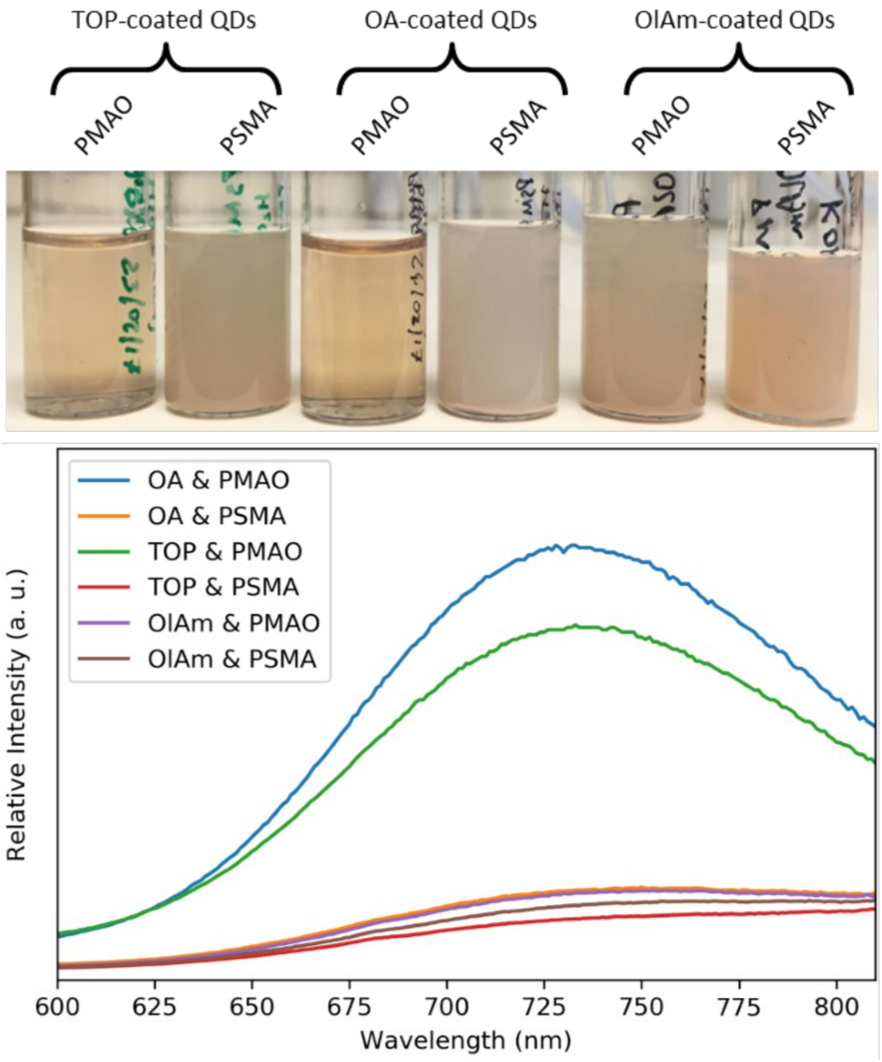

Figure S7. Top: an image of encapsulated QDs suspended in water, using either PSMA or PMAO derived amphiphilic polymers and trioctylphosphine (TOP), oleic acid (OA) or oleylamine (OAm) QD surface ligands. Bottom: the corresponding emission spectra for the encapsulated QD samples suspended in water. Excitation at 500 nm.

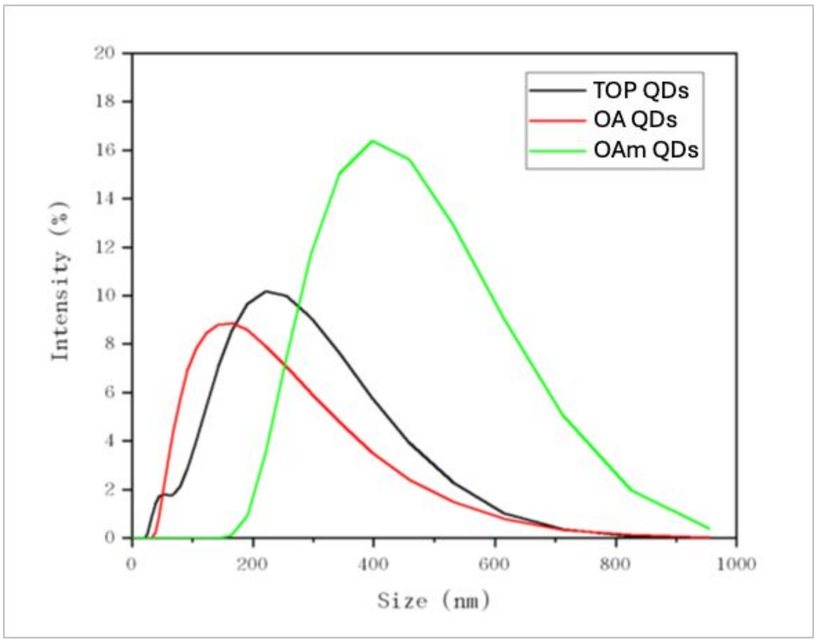

Figure S8. Dynamic light scattering intensity plot of three types of CuInZnS/ZnSe/ZnS particles capped with organic ligands.

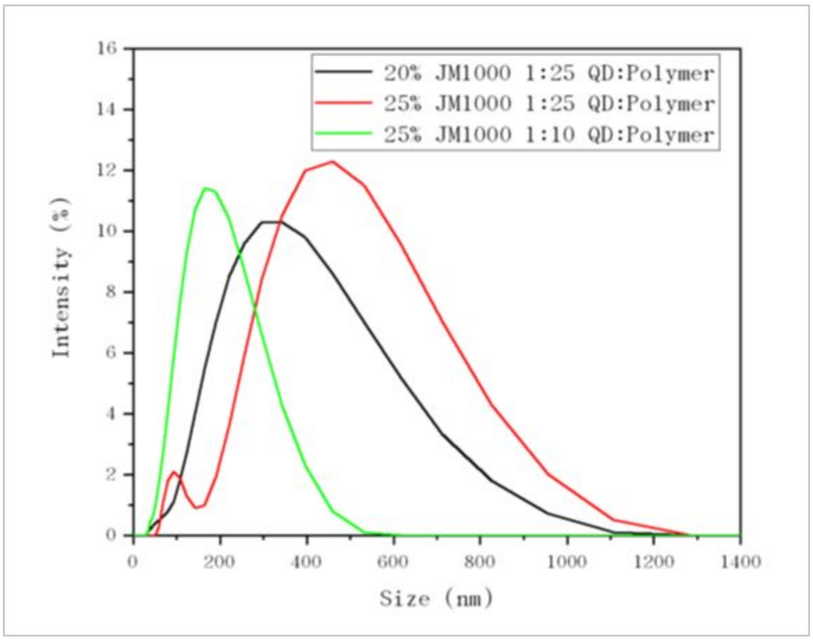

Figure S9. Dynamic light scattering intensity plot of CuInZnS/ZnSe/ZnS particles encapsulated with three different types of amphiphilic polymer.

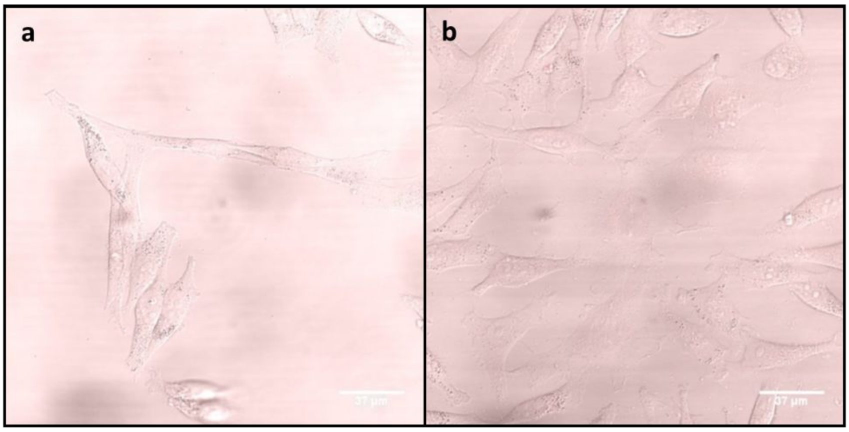

Figure S10. Light micrographs of HeLa cells incubated with QDs at 0.1 mg per mL, demonstrating cell morphology in the presence of QDs.

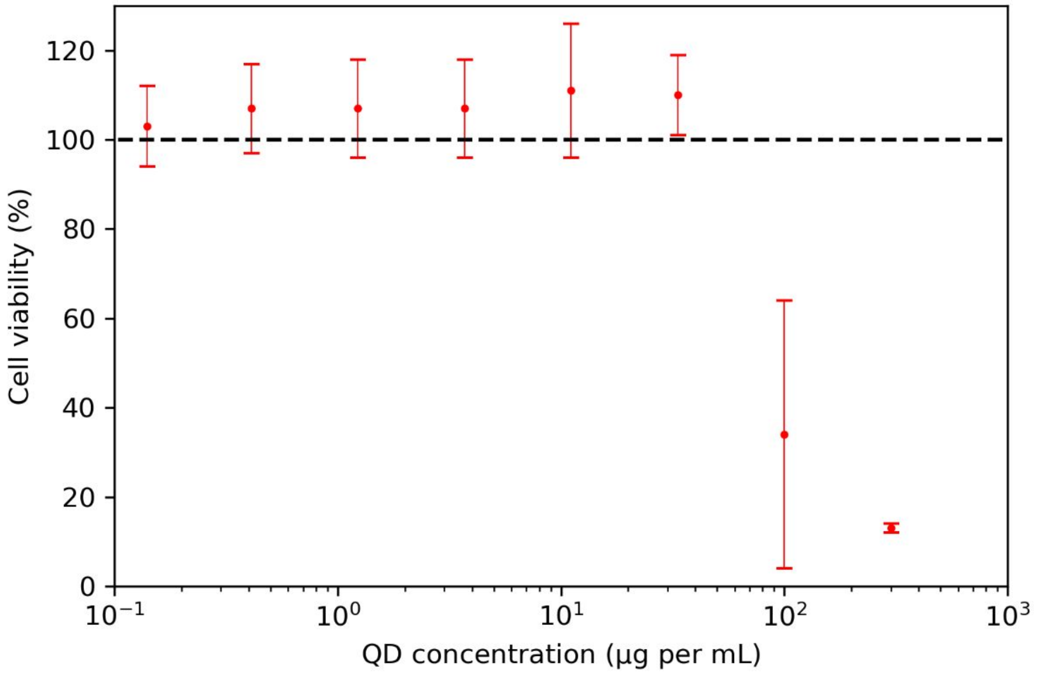

Figure S11. MTT assay data showing the viability of J447 murine cancer cells incubated with PMAO-Jeffamine M1000 encapsulated CuInZnS/ZnSe/ZnS QDs.

References

[1] Bowen Katari, J. E.; Colvin, V. L. Alivisatos, A. P. X-ray photoelectron spectroscopy of CdSe nanocrystals with applications to studies of the nanocrystal surface. *J. Phys. Chem*., **1994**, *98*, 4109 – 4117.

[2] Li, J. J.; Wang, Y. A.; Guo, W.; Keay, J. C.; Mishima, T. D.; Johnson, M. B.; Peng, X. Large-Scale Synthesis of Nearly Monodisperse CdSe/CdS Core/Shell Nanocrystals Using Air-Stable Reagents via Successive Ion Layer Adsorption and Reaction. *J. Am. Chem. Soc.*, **2003**, *125*, 12567–12575.

[3] Dong, B.; Cao, L.; Su, G.; Liu, W. Facile synthesis of highly luminescent UV-blue emitting ZnSe/ZnS core/shell quantum dots by a two-step method. *Chem. Commun.*, 2010, 46, 7331–7333.

[4] Tang, J.; Brzozowski, L.; Barkhouse, D. A. R.; Wang, X.; Debnath, R.; Wolowiec, R.; Palmiano, E.; Levina, L.; Pattantyus-Abraham, A. G.; Jamakosmanovic, D.; Sargent, E. H. Quantum Dot Photovoltaics in the Extreme Quantum Confinement Regime: The Surface-Chemical Origins of Exceptional Air- and Light-Stability," *ACS Nano*, **2010**, *4*, 869–878.

[5] Pons, T.; Pic, E.; Lequeux, N.; Casette, E.; Bezdetnaya, L.; Guillemin, F.; Marchal, F.; Dubertert, B. Cadmium-free $CuInS_2$/ZnS quantum dots for sentinel lymph node imaging with reduced toxicity. *ACS Nano*, **2010**, *4*, 2531–2538, 2010.

[6] Patterson, A. L. The Scherrer formula for X-ray particle size determination. *Phys. Rev.*, **1939**, *56*, 978–982.
